# mRNA dynamics and alternative conformations adopted under low and high arginine concentrations control polyamine biosynthesis in *Salmonella*

**DOI:** 10.1371/journal.pgen.1007646

**Published:** 2019-02-11

**Authors:** Tamar Ben-Zvi, Alina Pushkarev, Hemda Seri, Maya Elgrably-Weiss, Kai Papenfort, Shoshy Altuvia

**Affiliations:** 1 Department of Microbiology and Molecular Genetics, IMRIC, The Hebrew University-Hadassah Medical School, Jerusalem, Israel; 2 Munich Center for Integrated Protein Science (CIPSM) at the Department of Microbiology, Ludwig-Maximilians-University of Munich, Martinsried, Germany; Universidad de Sevilla, SPAIN

## Abstract

Putrescine belongs to the large group of polyamines, an essential class of metabolites that exists throughout all kingdoms of life. The *Salmonella speF* gene encodes an inducible ornithine decarboxylase that produces putrescine from ornithine. Putrescine can be also synthesized from arginine in a parallel metabolic pathway. Here, we show that *speF* expression is controlled at multiple levels through regulatory elements contained in a long leader sequence. At the heart of this regulation is a short open reading frame, *orf34*, which is required for *speF* production. Translation of *orf34* interferes with Rho-dependent transcription termination and helps to unfold an inhibitory RNA structure sequestering *speF* ribosome-binding site. Two consecutive arginine codons in the conserved domain of *orf34* provide a third level of *speF* regulation. Uninterrupted translation of *orf34* under conditions of high arginine allows the formation of a *speF* mRNA structure that is degraded by RNase G, whereas ribosome pausing at the consecutive arginine codons in the absence of arginine enables the formation of an alternative structure that is resistant to RNase G. Thus, the rate of ribosome progression during translation of the upstream ORF influences the dynamics of *speF* mRNA folding and putrescine production. The identification of *orf34* and its regulatory functions provides evidence for the evolutionary conservation of ornithine decarboxylase regulatory elements and putrescine production.

## Author summary

Polyamines are widely distributed in nature, they bind nucleic acids and proteins and although their exact mechanism of action is not clear, their effect on fundamental cellular functions is well documented. The canonical biosynthesis pathway of polyamines is conserved and begins with *speF* encoding ornithine decarboxylase, an inducible enzyme that produces putrescine from ornithine. Putrescine can also be produced from arginine in an alternative metabolic pathway. Here, we show that the rate of ribosome progression during translation of a short ORF (ORF34) upstream of *speF* influences the dynamics of *speF* mRNA folding and thus putrescine production. Uninterrupted translation of *orf34* carrying two consecutive arginine codons, under conditions of high arginine, results in the formation of a *speF* mRNA structure that is degraded by RNase G, whereas ribosomes slow-down at the consecutive arginine codons in the absence of arginine enables the formation of an alternative structure that is unsusceptible to RNase G and thus results in putrescine production. The study of *Salmonella speF* regulation provides evidence that, despite variations in the mechanistic details, RNA-based regulation of putrescine biosynthesis and ornithine decarboxylase is conserved from bacteria to mammals.

## Introduction

Regulatory RNAs are now recognized as important players in many adaptive and physiological responses in bacteria. RNA regulators allow bacterial cells to fine-tune their metabolism during growth [1], sense population density [2], modulate and modify cell-surface properties [3], and regulate virulence gene expression [4]. With respect to the molecular mechanisms involved, there are four main classes of regulatory RNAs in bacteria to date [5]. One extensively studied class are mRNA leaders, attenuators and riboswitches. This class of riboregulators control expression in *cis* by adopting altered structural conformations in response to cellular and/or environmental signals. For example, mRNA leaders of RNA thermosensors undergo significant changes in response to elevated temperatures [6] and RNA structural changes caused by stalled ribosomes affect transcription elongation through formation of terminator or antiterminator structures [7]. In contrast, riboswitches were defined as leader sequences that change their structure upon binding to small molecule ligands such as amino acids, carbohydrates, coenzymes and nucleobases [8, 9]. Riboswitches modulate gene expression at the level of transcription termination/elongation, translation initiation, or splicing [10, 11]. In many cases, this regulation involves the highly conserved transcription termination factor Rho [12]. For the majority of riboswitches, impaired translation promotes transcript termination by Rho. For example, translational repression of *thiMD* genes caused by the binding of TPP to *thiM* riboswitch promotes Rho-dependent premature termination in a region located between codons 20 and 34 of *thiM* [13]. A similar Rho-dependent transcriptional polarity was described for ChiX sRNA regulation of *chiPQ* operon. Inhibition of *chiP* translation by ChiX causes the nascent mRNA to become susceptible to Rho action, thereby preventing RNA polymerase from reaching *chiQ* [14]. The binding of Mg+2 and the FMN precursor to *mgtA* and *rib*, respectively, promotes structural changes in the RNA leader that facilitate interaction with Rho [15]. In case of the *mgtA* riboswitch, the leader *mgtL* also encodes a small, proline-rich peptide. Contrary to the canonical mechanism in which impaired translation favors Rho binding, complete translation of the *mgtL* peptide promotes association of Rho and premature termination [16, 17].

Searching for new riboswitches and other regulatory RNA motifs in α-proteobacteria, Corbino and colleagues [18] discovered a putative novel RNA element in the leader sequence of *speF*. The *speF* gene encodes an inducible ornithine decarboxylase catalyzing the initial step in putrescine production [19]. Putrescine can be produced from the precursor ornithine, via ornithine decarboxylase or from arginine through agmatine. Consequently, arginine and ornithine compete over the production of putrescine by being precursors of alternative pathways [20, 21]. Putrescine belongs to the large group of polyamines, an essential class of metabolites that exists throughout all kingdoms of life. In the absence of polyamines, a plethora of cellular processes are impaired, including gene expression, cell growth and/or proliferation and stress resistance [22]. The intracellular levels of polyamines are carefully controlled and so is the expression of ornithine decarboxylase, the limiting factor for putrescine biosynthesis. The expression of ornithine decarboxylase homologs are monitored at multiple levels, including transcription, translation and protein stability by highly conserved regulaory mechanisms involving feedback loops [22]. In mammals, translation of ornithine decarboxylase is repressed by high levels of polyamines via a small upstream ORF (uORF) encoded in a highly structured 5’UTR. The uORF and the long structured 5’UTR are conserved, regulating the expression of ornithine decarboxylase homologs in yeast, plants and mammals [22, 23].

Here we show that *Salmonella speF-potE* operon carries a long mRNA leader, which encodes an ORF of 34 amino acids (here denoted *orf34*). Regulatory elements encoded within the mRNA leader including two consecutive arginine codons facilitate *speF* expression control at multiple levels including premature transcription termination, translation initiation and mRNA decay.

## Results

### Translation of *orf34* prevents premature transcription termination by Rho

**Fig 1:**
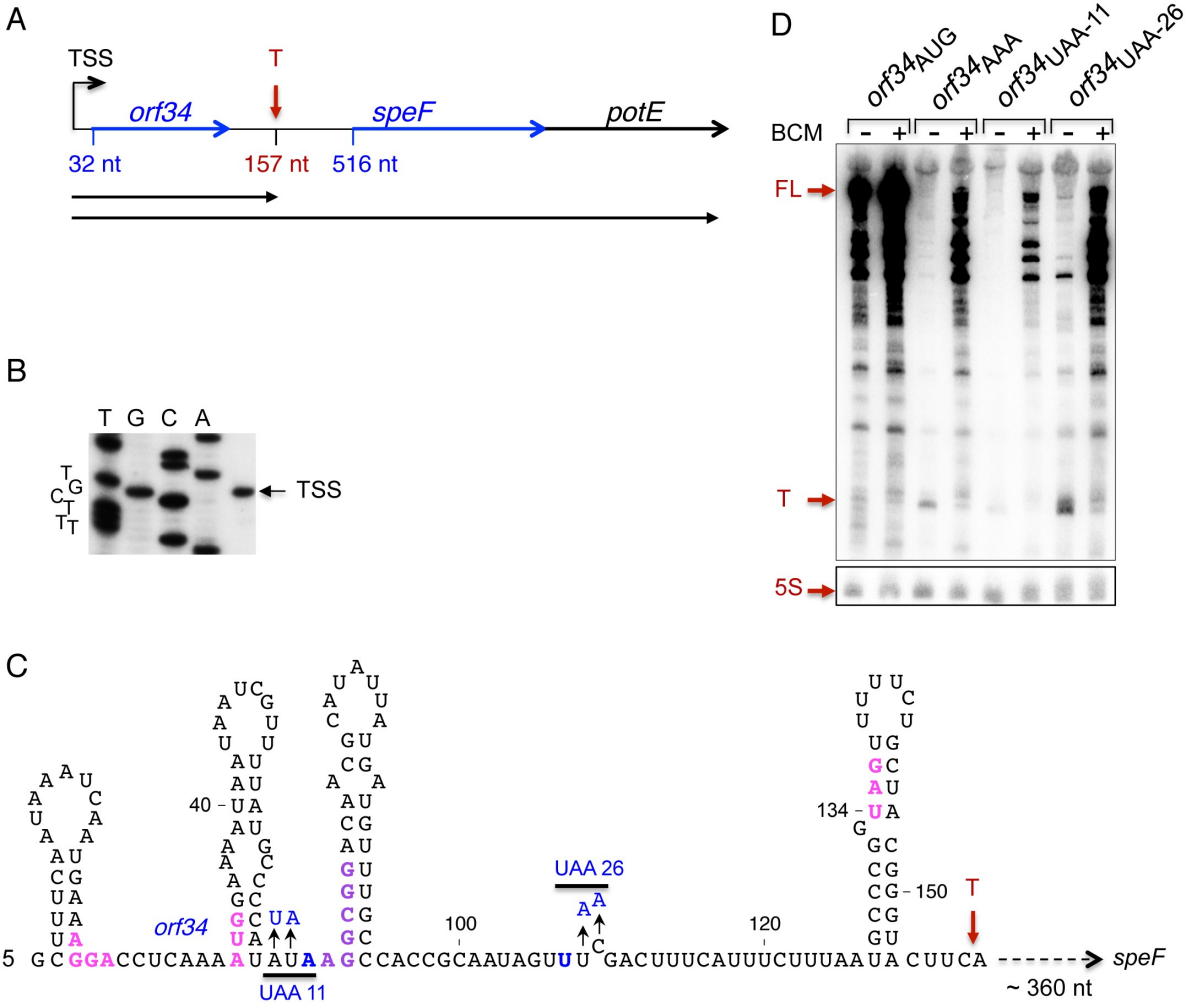
Characteristics of the *speF* locus. (A) Schematic representation of the *speF* locus including *orf34*, *speF* and *potE*. The transcription start site (TSS) is indicated. The initiation codons of *orf34* and *speF* are located at nucleotides 32 and 516 of the mRNA, respectively. The red vertical arrow indicates the site of *rho*-dependent premature transcription termination (T). Horizontal arrows indicate the short transcript obtained due to Rho (157 nt) and the full-length polycistronic mRNA. (B) Start site determination. The transcription start site of *speF* operon was determined by primer extension using primer 1614. RNA was extracted form cultures grown in LB to OD600 of 0.5. (C) The sequence of the transcript obtained by premature termination. The Shine-Dalgarno (AGGA), the initiation codon (AUG) and the stop codon (UGA) of *orf34* are in magenta. The consecutive arginine codons AGG;CGG are in purple. UAA11 and UAA26 indicate mutated codons leading to premature translation termination and thus to premature transcription termination by Rho. (D) Translation of the entire *orf34* prevents premature transcription termination by Rho. Cultures carrying P*tac-orf34-speF* plasmids wild type and translational mutants as indicated (*orf34*AAA; *orf34*UAA11 and *orf34*UAA26), were grown in LB to OD600 of 0.2 and then treated with 20 μg/ml BCM for 60 minutes (+) or were left untreated (-). Northern blot of RNA samples (10 μg) separated using 6% urea-polyacrylamide gels. The membranes were probed with end-labeled *orf34* (1614) and 5S rRNA (459) specific primers. 5S RNA serves as a loading control. The Full-length (FL) and the short transcript obtained by premature termination (T) are indicated in red.

To investigate a possible role of the 5’UTR in post-transcriptional regulation of *speF* in *Salmonella*, we first mapped the transcriptional start site (TSS) using primer extension. We discovered only a single initiation site located 516 nucleotides upstream of the *speF* ORF (Fig 1A and 1B), which is in line with previous reports [24]. A *speF*-*lacZ* transcriptional reporter revealed that the gene is transcribed in rich media and that deletion of the *speF* promoter strongly inhibited expression (Table 1). The long 5’UTR of *speF* prompted us to explore its sequence. The search revealed a short putative ORF of 34 amino acids (*orf34*) encoded close to the transcription start site, at position +32 of the mRNA (Fig 1A). Transcription and translation reporter fusions demonstrated that ORF34 is efficiently translated. Replacing the initiation codon of *orf34* with AAA abolished translation of *orf34-lacZ* fusion (Table 1; rows 4,5). We used this mutant to examine the influence of *orf34* translation on *speF* expression. Lack of *orf34* translation strongly reduced *speF*-*lacZ* expression; both the transcription and the translation of *speF-lacZ* decreased dramatically (Table 2; rows 1,2). Furthermore, premature translation termination caused by replacing amino acids 11 and 26 of *orf34* with stop codons (*orf34*UAA11 and *orf34*UAA26 respectively) weakened expression of *speF-lacZ* to a similar extent, indicating that expression of *orf34* is required for transcription of the *speF* gene (Fig 1C and Table 2).

**Table 1:** Transcription and translation at the *orf34-speF* locus.

Genotype^a^	Transcription fusion	Translation fusion
1. P-*orf34-speF-lacZ*	5263±820	7081±1491
2. ΔP-*orf34-speF-lacZ*	65 ± 7	5 ± 2
3. pRS551/pRS552	10 ± 2	3 ± 2
4. P-orf34-lacZ*	145 ± 6	4977 ± 130
5. P-*orf34*AAA-lacZ*	115 ± 29	3 ± 2

^a^The *orf34-speF* fusions harbor the promoter region (P), *orf34* and *speF* fused to *lacZ in* PRS551 (transcription) and PRS552 (translation) fusion vectors. In ΔP-*orf34-speF-lacZ*, the promoter has been deleted. In P-*orf34-lacZ*, the *orf34* is directly fused to *lacZ*. Where indicated, the initiation codon of *orf34* (AUG) has been replaced by AAA (*orf34*AAA).

*Because of the high LacZ activity of *orf34-lacZ* translation fusion, all *orf34* fusions were inserted in the chromosome as a single copy. The cultures were grown in LB to OD600 of 0.9. Results are displayed as mean of 2 colonies ± standard deviation.

**Table 2:** Translation of *orf34* prevents premature termination caused by Rho.

Genotype^a^	Transcription fusion		Translation fusion	
	-BCM	+BCM	-BCM	+BCM
1. P-*orf34*AUG-*speF-lacZ*	5263±820	5413 ± 171	7081±1491	5116 ± 125
2. P-*orf34*AAA-*speF-lacZ*	53 ± 4	1762 ± 176	1 ± 6	82 ± 15
3. P-*orf34*UAA-11-*speF-lacZ*	61 ± 21	3216 ± 1407	4 ± 4	169 ± 56
4. P-*orf34*UAA-26-*speF-lacZ*	72 ± 3	3635 ± 1139	5 ± 1	135 ± 43

^a^The *orf34-speF* fusions harbor the promoter region (P), *orf34* and *speF* fused to *lacZ*. In *orf34*AAA, the initiation codon of *orf34* was replaced by AAA. In *orf34*UAA-11 and *orf34*UAA-26, premature stop codons (UAA) were inserted in *orf34* at amino acid positions 11 and 26, respectively. Cultures grown in 7 ml LB medium to OD600 of 0.2 were treated with 20μg/ml of Bicyclomycin for 60 min. Results are displayed as mean of 2 colonies ± standard deviation.

The ~100-fold reduction in activity of the transcriptional reporter (5263±820 to 53±4, Table 2) prompted us to examine the pattern of RNA species in wild type and *orf34* translational mutants. To this end, plasmid-borne expression of the *orf34*-*speF* locus (P*tac*-*orf34-speF*) carrying wild type and *orf34* translational mutants was monitored using Northern Blots. The data in Fig 1D show that the translational mutants (*orf34*AAA; *orf34*UAA11 and *orf34*UAA26) produced mainly a short RNA species of ~157 nucleotides (indicated by T) as determined by 3’-end RACE. Therefore, in the absence of *orf34* translation or upon premature translation termination, the transcription terminates before it reaches the *speF* coding-region. Given that sequence analysis of the *speF* 5’UTR did not reveal a *rho*-independent transcription terminator, we speculated that in the absence of *orf34* translation, the transcription termination factor Rho terminated elongation. We tested this hypothesis by using bicyclomycin (BCM), which inhibits the ATPase activity of Rho [25]. Northern Blot analysis demonstrated that upon addition of BCM, transcription of *orf34*-*speF* (P*tac*-*orf34-speF*) carrying *orf34* translational mutants (AAA, UAA11 and UAA26) extended into the *speF* ORF (Fig 1D). Likewise, the levels of the *orf34-speF-lacZ* transcriptional fusions carrying *orf34* mutations increased with BCM, indicating that complete *orf34* translation is required to prevent premature transcription termination by Rho (Table 2; transcription fusion +BCM). Moreover, as UAA-26 includes RNA species that result from escaped transcription elongation, the closer the site of the stop codon is to the end of the peptide, the lower the activity of Rho.

### Translation of *orf34* facilitates downstream translation of *speF*

**Fig 2:**
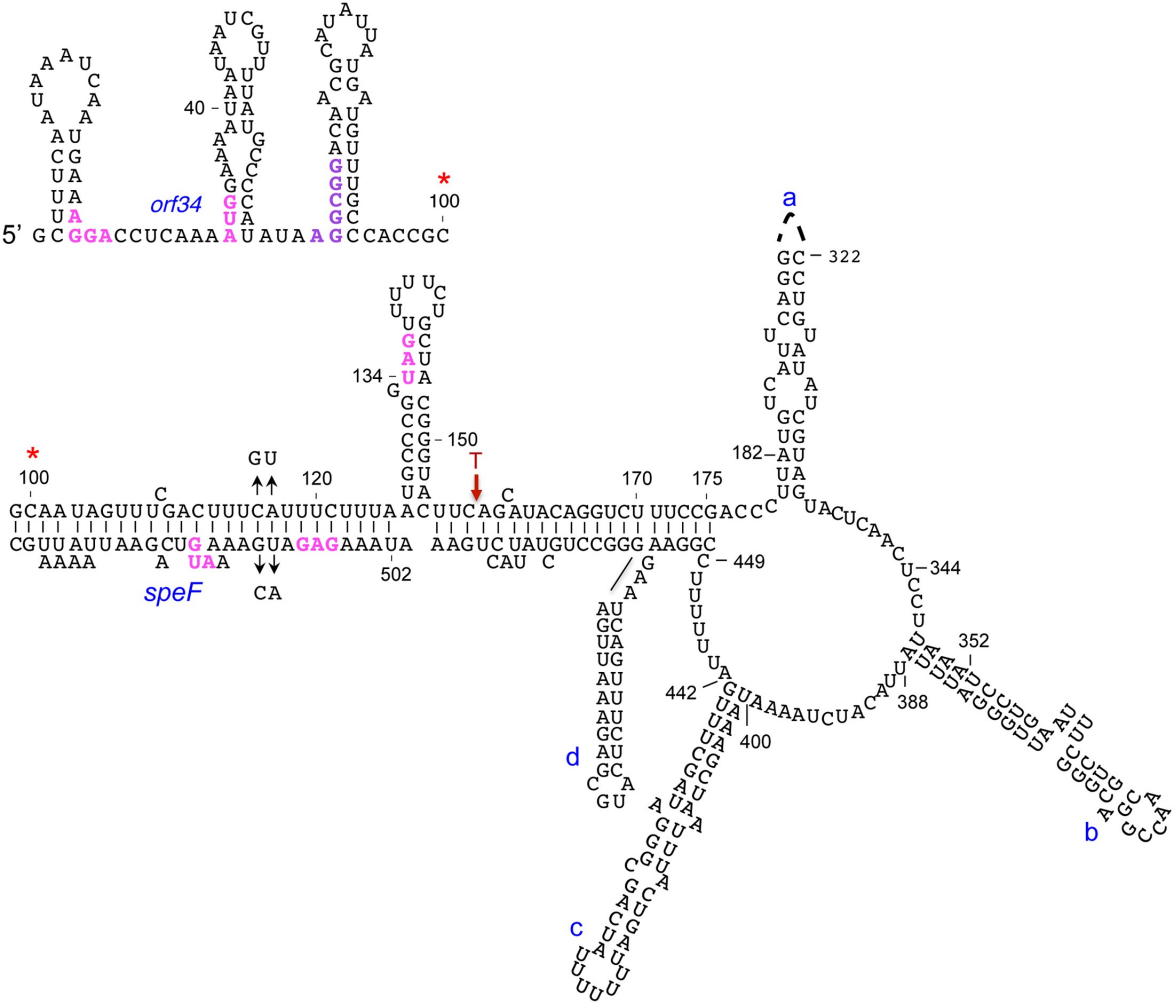
Putative long-range interaction at the *speF* locus as predicted by RNAfold. The sequence starts at the upper left side of the slide and the red asterisk indicates its continuation. The Shine-Dalgarno sequences of *orf34* and *speF*, both AUGs and *orf34* stop codon are indicated in magenta. The structure predicted harbors a ring-like structure encompassing nucleotides 175 to 449 followed by a long helix in which nucleotides 99–125 of *orf34* basepair with nucleotides 502–535 overlapping the ribosome binding site of *speF* and nucleotides 155–175 upstream of *orf34* basepair with nucleotides 450 to 454 and 482 to 500 upstream of ribosome binding site of *speF*. Mutations that disrupt the helix and compensatory mutations that restore helix-formation are indicated by arrows (GU at position 116,117 and CA at position 510,511). Premature transcription termination site by Rho is indicated (T).

The data presented in Table 2 showing that the activity of the *orf34*AAA-*speF-lacZ* transcriptional reporter increased with BCM, whereas the levels of the corresponding translation fusion remain low, indicated that unlike the ribosome-binding site (RBS) of *lacZ*, the RBS of *speF* mRNA is translationally inactive under these conditions. These results suggest that translation of *orf34* has a positive effect on *speF* translation initiating approximately 500 nts downstream and motivated a search for potential long-range RNA interactions. The RNA-fold program [26] predicted such a potential long-range interaction covering the 5’-end of the transcript and the sequence surrounding the RBS of *speF* (Fig 2). The predicted structure contains a ring-like organization encompassing nucleotides 175 to 449 followed by a long helix in which nucleotides 99–125 of *orf34* base-pair with nucleotides 502–535 overlapping the ribosome binding site of *speF* and nucleotides 155–175 upstream of *orf34* base-pair with nucleotides 450 to 454 and 482 to 500 upstream of the ribosome binding site of *speF* (Fig 2). To validate basepairing around the RBS of *speF*, we exchanged nucleotides cytidine 116 and adenine 117 to guanine and uracil, respectively in *orf34* and uracil 510 and guanine 511 with adenine and cytidine on the opposite strand. These mutations when combined together are predicted to restore helix formation. To examine basepairing *per se*, we prevented upstream translation using *orf34*AAA, while allowing transcription elongation into *speF* using BCM. The translation fusions of these mutants demonstrated that disruption of hybrid formation increased *speF* translation (Table 3). Furthermore, using the corresponding complementary mutations restoring basepairing resulted in decreased *speF* translation (Table 3). Together, our data demonstrate that upstream translation of *orf34* prevents premature termination upstream of *speF* and facilitates translation of the *speF* mRNA by disrupting an inhibitory RNA structure sequestering the RBS of *speF*.

**Table 3:** Translation of *orf34* facilitates translation of *speF*.

Genotype^a^	Translation fusion +BCM
P-*orf34*AAA-*speF-lacZ*	95 ± 33
ΔP-*orf34*AAA C116G;A117U-*speF-lacZ*	1037 ± 202
ΔP-*orf34*AAA U510A;G511C-*speF-lacZ*	309 ± 74
ΔP-*orf34*AAA C116G;A117U, U510A;G511C -*speF-lacZ*	66 ± 11

^a^The *orf34-speF* fusions harbor the promoter region (P), *orf34* and *speF* fused to *lacZ*. All fusions carry *orf34*AAA mutation in addition to the mutations that prevent formation of the hybrid as mentioned. BCM was added at OD600 of 0.2 for 1 hour. Results are displayed as mean of 4 colonies ± standard deviation

### Positive and negative regulation of *speF* by ornithine and arginine, respectively

**Fig 3:**
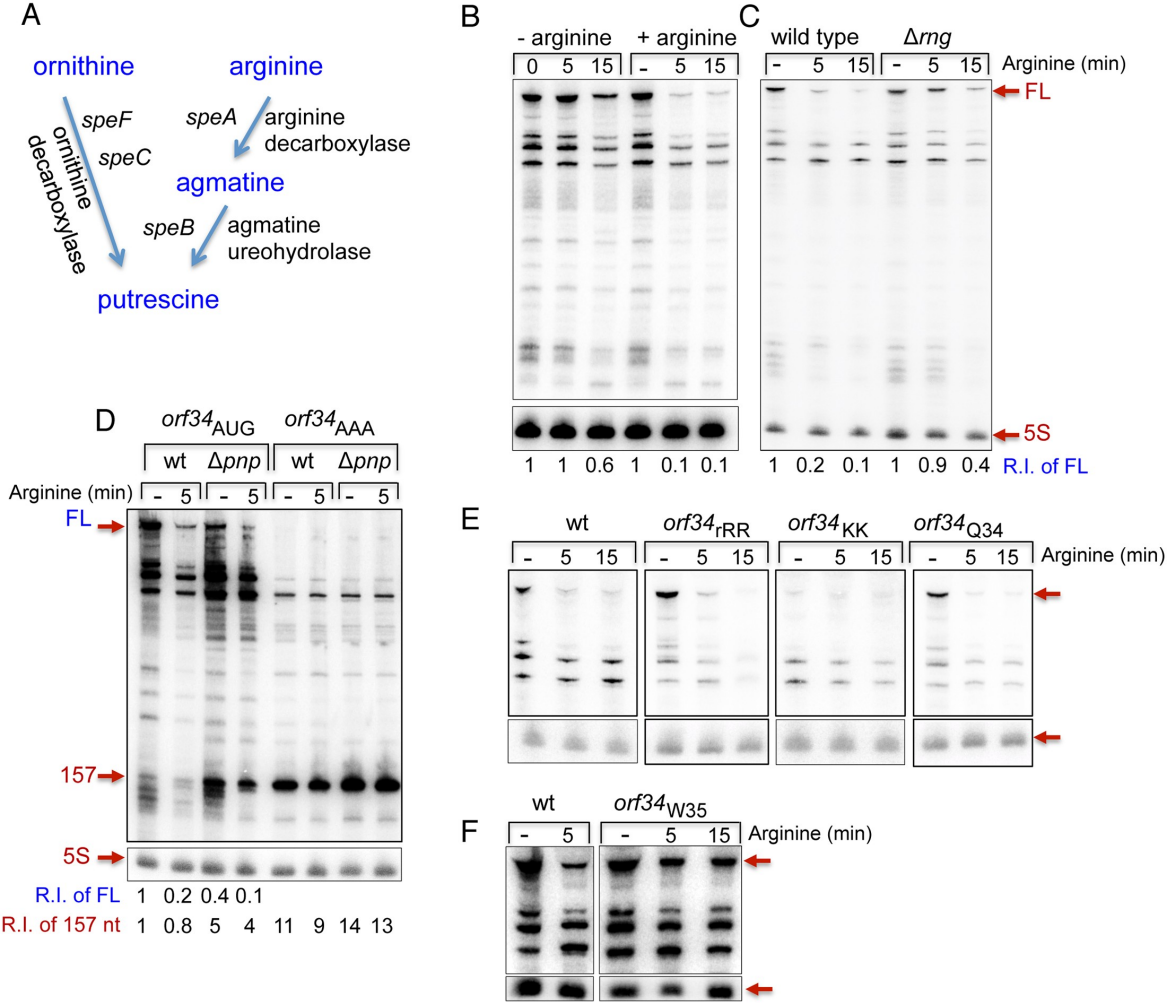
Arginine-dependent mRNA destabilization. (A) A partial diagram of polyamine biosynthesis. Putrescine can be produced from ornithine via *speC* and *speF* encoding a constitutive and inducible ornithine decarboxylase, respectively or from arginine in two steps via *speA* and *speB* encoding arginine decarboxylase agmatine ureohydrolase respectively. (B) *speF* mRNA levels decrease in the presence of arginine. Cultures of wild type carrying P*tac-orf34-speF* were grown in E-minimal media to OD600 of 0.2 and then treated with arginine (100 μg/ml) or left untreated for the indicated time. Northern blots as in Fig 1D. Relative intensity of the full-length RNA measured by ImageQuant TL 1D v8.1 is shown (R.I of FL). (C) RNase G destabilizes the full-length mRNA in the presence of arginine. Cultures of wild type and RNase G mutant (Δ*rng*) carrying P*tac-orf34-speF* were grown in E-minimal media to OD600 of 0.2 and then treated with arginine (100 μg/ml) for the indicated time. Relative intensity of the full-length RNA vs. 5S is shown. RNA samples taken from cells at OD600 of 0.2 before exposure to arginine are indicated as (-). (D) The 3’-end exonuclease PNPase degrades the short transcript produced by premature termination. Cultures of wild type and PNPase mutant (Δ*pnp*) carrying P*tac-orf34-speF* and P*tac-orf34*AAA-*speF speF* were grown in E-minimal media to OD600 of 0.2 and then treated with arginine (100 μg/ml) for the indicated time as described in C. Relative intensity of the 157 nt long transcript measured by ImageQuant TL 1D v8.1 is shown (R.I of 157 nt). The short transcript accumulates in Δ*pnp* mutant (5 fold). Unlike the 157 nt transcript, the full-length transcript (R.I of FL) decreases by 2 fold in Δ*pnp* compared to wild type. (E) The regulation by arginine depends on the arginine codons at positions 12 and 13 but not on the arginine codon at position 34. Cultures with P*tac-orf34-speF* carrying mutations in the arginine codons as indicated were grown and treated as described above. The Northern blot was probed with end-labeled *orf34* (2411) and 5S rRNA (459) specific primers. (F) mRNA destabilization by arginine depends on the position of the stop codon. Cultures carrying wild type P*tac-orf34-speF* and P*tac-orf34*W35-*speF* in which the stop codon of *orf34* was replaced with Trp codon were grown and treated as described above. The Northern blot was probed with end-labeled *orf34* (2411) and 5S rRNA (459) specific primers.

Putrescine can be produced from its precursor ornithine by *speF* encoding ornithine decarboxylase, an inducible enzyme, or from arginine in a metabolic pathway that involves both arginine decarboxylase (*speA*) and agmatine ureohydrolase (*speB*) (see diagram in Fig 3A). Whereas, ornithine is converted directly to putrescine, arginine is first converted to agmatine and subsequently to putrescine [20, 21]. We tested the effect of ornithine and arginine on *speF* expression using *orf34-speF-lacZ* fusions. The activity of *orf34*-*speF-lacZ* increased in minimal medium supplemented with ornithine (Table 4 row 1) and decreased in medium supplemented with arginine (Table 4 row 4). To determine which of the genetic elements mediated the effect of the precursors, we examined activity of the *speF* promoter (P-*speF*-*lacZ*) and *orf34* (P-*orf34-lacZ*) in the presence and absence of ornithine and arginine. Our data revealed that neither the *speF* promoter, nor *orf34* were influenced by ornithine or arginine (Table 4 rows 2,3 and 5,6). In summary, our results show that ornithine induces expression of ornithine decarboxylase, however in presence of the alternative precursor arginine, the inducible pathway producing putrescine via *speF* is turned off.

**Table 4:** Regulation of *orf34* and *speF* by ornithine and arginine (β-galactosidase activity).

Genotype^a^	Transcription fusion			Translation fusion	
	-Ornithine	+ Ornithine	Control	- Ornithine	+ Ornithine
1. P-*orf34*AUG-*speF-lacZ*	908 ± 35	5624 ± 760	+	900 ± 351	6777 ± 1666
2. P-lacZ*	3047 ± 51	2721 ± 175	-		
3. P-orf34-lacZ*	482 ± 70	414 ± 22	-	17466 ± 3347	16121 ± 2883
	**-Arginine**	**+Arginine**		**-Arginine**	**+Arginine**
4. P-*orf34*AUG-*speF-lacZ*	908 ± 35	281 ± 92	+	900 ± 351	33 ± 30
5. P-lacZ*	2182 ± 112	2050 ± 92	-		
6. P-orf34-lacZ*	385 ± 60	433 ± 33	-	15052 ± 4641	14612 ± 5508
7. P-*orf34*rRR -*speF-lacZ*	465 ± 52	251 ± 47	+	213 ± 104	20 ± 20
8. P-*orf34*fRR-*speF-lacZ*	1015 ± 123	410 ± 91	+	860 ± 290	99 ± 40
9. P-*orf34*KK-*speF-lacZ*	243 ± 53	239 ± 31	-	18 ± 2	9 ± 2
10. P-*orf34*Q34-*speF-lacZ*	857 ± 137	180 ± 121	+	582 ± 267	133 ± 124
11. P-*orf34*W35-*speF-lacZ*	2577 ± 423	1917 ± 210	-	2277 ± 326	1809 ± 422

^a^The *orf34-speF* fusions harbor the promoter region (P), *orf34* and *speF* fused to *lacZ*. In P-*orf34-lacZ*, the *orf34* is directly fused to *lacZ*. *Because of the high LacZ activity of the translation fusion *orf34-lacZ*, all *orf34* fusions were inserted in the chromosome as a single copy. In P-*lacZ* the promoter of *speF* was directly fused to *lacZ* and the fusion was inserted in the chromosome as a single copy. In *orf34*rRR, *orf34*fRR and *orf34*KK, the two rare arginine codons were replaced by other identical rare arginine, frequent arginine and Lys codons, respectively. In *orf34*Q34 the rare arginine codon was replaced by Gln codon. In *orf34*W35 the stop codon was replaced by Trp codon. The *orf* was extended by 23 amino acids till it reached a new stop codon. Fusions regulated (+) and unregulated (-) by the precursors. Results are displayed as mean of 3 (P-lacZ*), 5–26 (all others) colonies ± standard deviation. Cultures were grown for 17 hours from a single colony in 5 ml (50 ml tubes) of E Minimal supplemented with ornithine or arginine (100 μg /ml).

### Two consecutive arginine codons in *orf34* mediate *speF* regulation by arginine

Exploring the *orf34* sequence, we found that ORF34 homologues are conserved among γ-proteobacteria carrying a highly conserved domain of unknown function 2618 (DUF2618; pfam: PF10940) (S1 Fig). The core of DUF2618 is conserved carrying two consecutive arginine residues (S1 Fig: HIRRT*HIMM). In *Salmonella*, the coding sequence of DUF2618 core harbors two consecutive, rare arginine codons (AGG and CGG) in the 5’-end of *orf34* and an additional one (CGG) adjacent to its stop codon (Fig 1C). The conservation of the arginine codons as well as of the position of ORF34 homologues *i*.*e*. in front of *speF* prompted us to hypothesize that ribosome stalling at these codons alters the structure of the *speF* mRNA and that this structural change is required for down-regulation of *speF*. To investigate if the arginine codons are involved in arginine sensing, we changed the consecutive rare arginine codons with either two other rare arginine codons (CGACGA; rRR), with frequent arginine codons (CGCCGC; fRR), or with lysine codons (AAGAAG; KK). *orf34-speF*-*lacZ* carrying consecutive other rare (rRR) or frequent (fRR) arginine codons was regulated by arginine similar to wild type, indicating that arginine codons either rare or frequent mediate the regulation by arginine (Table 4). Furthermore, changing these codons with lysine codons abolished *speF* regulation by arginine. Changing the rare arginine codon positioned adjacent to the stop codon, with Gln (Q34) had no effect on *speF* expression regulation by arginine (Table 4; rows 4, 7–10). The conserved core amino acids of ORF34 also prompted us to examine whether translation of ORF34 in *trans* affects expression of *speF*. The activity of transcription and translation fusions of P-Δ(*orf34*)-*speF-lacZ* and P-*orf34*AAA-*speF-lacZ* was unaffected by arginine when ORF34 was expressed in *trans* (S1 Table). Together, the data indicate that two consecutive arginine codons at positions 12 and 13 independent of whether rare or frequent sense and transduce the signal of arginine availability.

### *speF* mRNA destabilization by Arginine

To characterize the effect of arginine on *speF* expression, we analyzed *speF* mRNA levels in the absence and upon addition of arginine. The northern shows that exposure of cells to arginine led to about 10-fold decrease in *speF* mRNA within the first 5 minutes of treatment while *speF* mRNA of untreated cell was stable (Fig 3B). Given that arginine had no effect on promoter activity of *speF* (Table 4 row 5) and did not induce premature transcription termination (Fig 3B), we concluded that arginine turns off *speF* pathway by affecting *speF* mRNA stability. To determine which ribonucleases are involved in *speF* mRNA decay, we examined the effects of *rne*, *rng* and *pnp* encoding endoribonuclease E, endoribonuclease G and 3’-end exoribonuclease PNPase, respectively. Analysis of the RNA levels upon exposure to arginine in wild type and in *rng* mutant revealed a significant decrease in *speF* mRNA in wild type while the decrease in *speF* mRNA in Δ*rng* cells was minimal, indicating that endoribonuclease G is involved in *speF* mRNA decay (Fig 3C). Kinetics studies to monitor the decrease in *speF* mRNA levels upon exposure of arginine showed that the levels of this mRNA decreased rapidly in wild type cells reaching ~10% of its initial level by 10 min of exposure, whereas in *rng* mutant *speF* RNA decay was significantly slow reaching ~ 40% of its initial levels by 10 min of exposure (S2 Fig). Mapping of RNase G cleavage sites by primer extension of RNA samples extracted from untreated wild type and Δ*rng* cells and samples of cells exposed to arginine for 2 and 10 min revealed a number of cleavage sites at 10 min of exposure at which mRNA decay was more prominent. Notably, whereas a few of the sites may have resulted from secondary cleavage, the cleavage sites detected within the ring structure were specific to adenines reminiscent of the RNase E consensus site (G/A N↓A/U U/C U/A) [27] (S3 & S4 Figs). Unlike *rng*, the full length of *speF* mRNA was not detected in a temperature sensitive mutant of RNase E (S5 Fig). It is possible that in the absence of RNAse E other ribonucleases took over this function.

RNA analysis of *speF* mRNA (*orf34*AUG) in wild type and Δ*pnp* demonstrates that the 3’ exoribonuclease PNPase affects the stability of the 157 nt long RNA species as well as of RNA species obtained by degradation upon exposure to arginine (Fig 3D). Quantification of the levels of the 157 nt transcripts in wild type vs. Δ*pnp* shows that the transcript produced by *orf34*AUG is ~5 fold higher in Δ*pnp* compared to wild type (Fig 3D). These results indicate that a significant portion of the *speF* transcript produced under conditions of low and high arginine terminates prematurely and that the terminated transcript is quickly degraded and thus its abundance is reduced in wild type cells (Fig 3D). The truncated transcript produced by *orf34*AAA is only slightly higher (1.35 fold) in Δ*pnp* compared to wild type. However, the basal level of the transcript produced by *orf34*AAA in wild type cells is ~10 fold higher than that of the transcript produced by *orf34*AUG. Thus, we suspect that its degradation by PNPase could be masked by other 3’-end exoribonucleases such as RNase II or RNase R.

In addition, we examined the effect of arginine on *speF* mRNA carrying rare arginine codon mutants (*orf34*rRR and *orf34*Q34). The northern showed that similarly to wild type, and as indicated by the *lacZ* fusions, *orf34*rRR-*speF* and *orf34*Q34-*speF* mRNAs produced by P*tac*-*orf34-speF* were destabilized by arginine (Fig 3E). Intriguingly, only low levels of full-length mRNA were detected in *orf34*KK-*speF* in which the consecutive arginine codons were changed with lysine, indicating that translation of *orf34* in the absence of the arginine codons at positions 12 and 13 results in transcript degradation (Fig 3E). Likewise, wild type *orf34* translation through the consecutive arginine codons under conditions of high arginine results in mRNA degradation.

### The position of the stop codon of *orf34* affected regulation by arginine

Our data suggested that complete translation of the *orf34* results in transcript destabilization and prompted us to examine whether the position of the stop codon of *orf34* affected regulation by arginine. By replacing the stop codon at the end of *orf34* with Trp codon (W35), *orf34* was extended by 23 amino acids when reaching a new stop codon. Transcription and translation fusion levels of *orf34*W35-*speF-lacZ* demonstrate that moving the position of the stop codon further downstream abolishes regulation by arginine (Table 4 compare lanes 1 and 11). Northern Blot analysis showed that the mutant is no longer subjected to arginine regulation, as the transcript remains stable in the presence of arginine (Fig 3F). Therefore, the position of the stop codon together with full translation of *orf34* to its native stop codon determines transcript stability.

### Formation of an alternative structure

**Fig 4:**
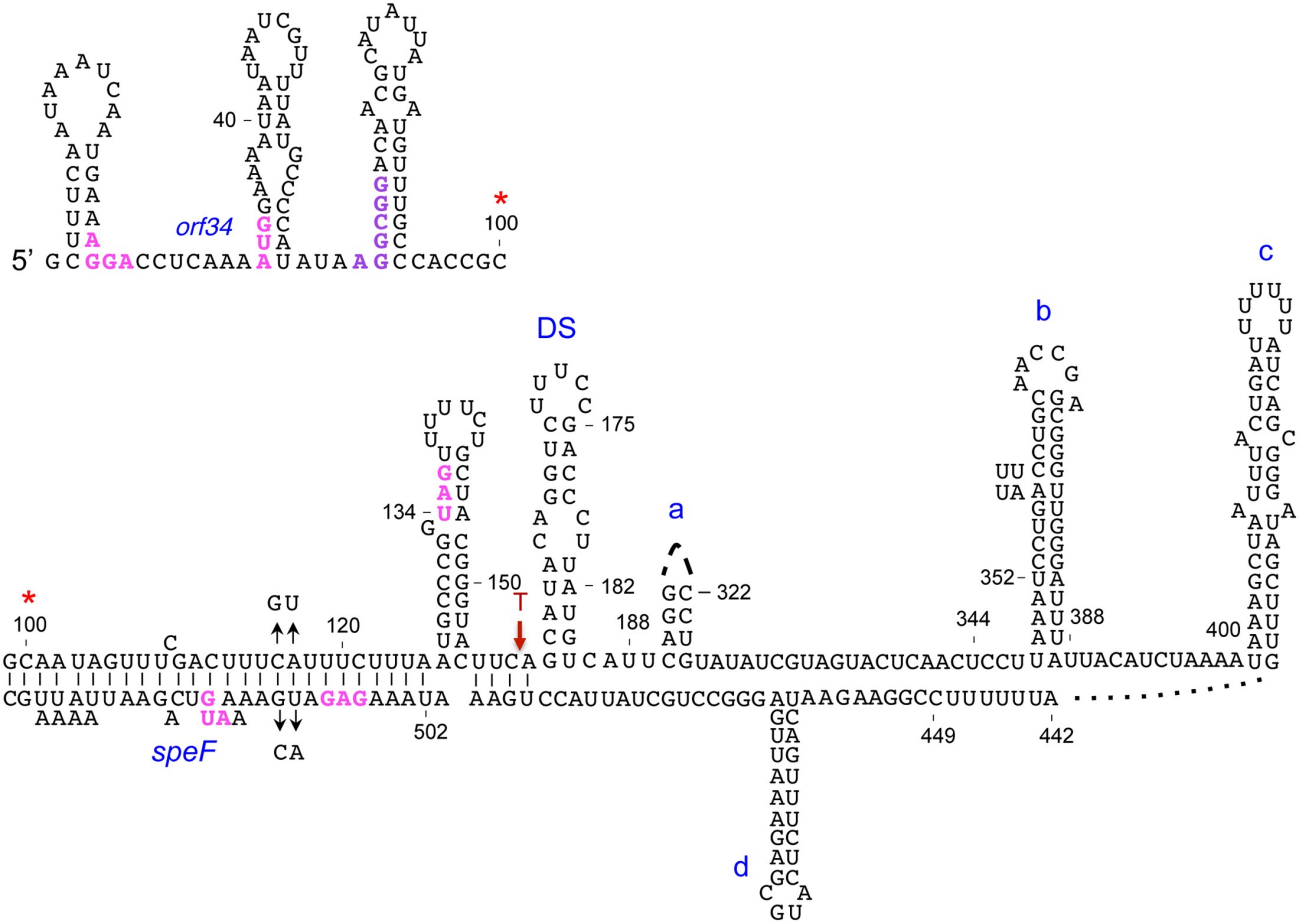
Putative alternative structure predicted to form under low arginine. The sequence starts at the upper left side of the slide and the red asterisk indicates its continuation at the lower part of the slide. In this structure the hairpin downstream of stop (DS) is formed, preventing formation of the ring-like structure and of the helix engaging nucleotides 155–175 and 450–500 (see also Fig 2). The Shine-Dalgarno sequence of *orf34*, the AUG and *orf34* stop codon are indicated in magenta. The consecutive arginine codons AGG CGG are in purple.

**Fig 5:**
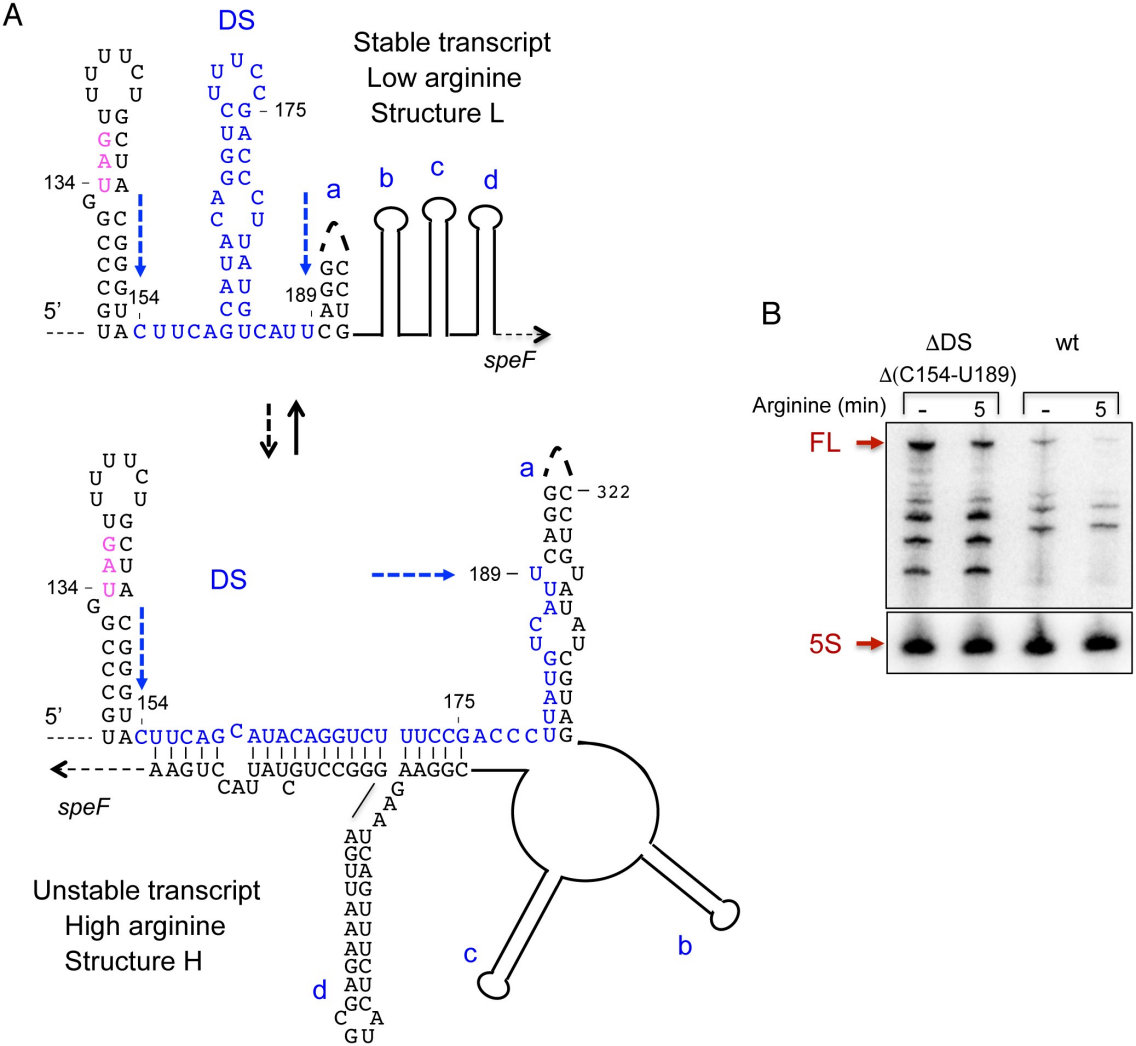
Deleting hairpin DS prevents formation of the ring-like structure and the RNA remains stable. (A) Structures predicted to form under low and high arginine conditions (L and H, respectively) are shown. The sequence of hairpin DS is marked in blue. (B) RNA analysis of P*tac-orf34-speF* wild type and deletion mutant missing nucleotides C154 to U189 with and without arginine as indicated.

The observations that *orf34-speF* locus can produce both stable and unstable transcripts and that ribosome pausing due to amino acid deficiency affect *speF* mRNA decay indicated that the *speF* mRNA could form alternative RNA structures. As the final result of RNA folding algorithms is typically different from the RNA structures occurring during the continuous process of transcript elongation during transcription, we searched for alternative structures using shorter sequences. The RNAfold program consistently predicted formation of a new hairpin right downstream of the hairpin encompassing *orf34* stop codon (Fig 4). We hypothesized that formation of a hairpin structure downstream of the stop codon (DS) prevented annealing of the proximal strand of this hairpin with a sequence downstream of the ring-like structure, while the distal strand could no longer be part of the helix a. Thus, formation of hairpin DS would interfere with the formation of the alternative structure (Structure H, Fig 5A). To test this hypothesis, we deleted the DS sequence and investigated RNA stability. Specifically, deleting this sequence is unlikely to affect the structure formed under low arginine conditions (Structure L, Fig 5B), but would prevent formation of the structure predicted to form under high arginine conditions (Structure H) (Fig 5A). Northern Blot analysis of ΔDS (ΔC154-U189) with and without arginine revealed significantly increased RNA levels when compared to the wild type construct, indicating that hairpin DS plays a role in the formation of the unstable structure H and that the stable L structure forms under low arginine conditions (Fig 5B).

The results showing that RNAs produced by the mutants; *orf34*W35-*speF* and ΔDS are stable and unaffected by arginine suggest that ribosomes pausing at the natural position of *orf34* stop codon lead to the formation of the metabolically unstable alternative structure via destabilization of the DS hairpin. *In vivo* structure probing of the stop codon hairpin and the neighboring downstream DS hairpin of wild type and *orf34*W35-*speF* showed that wild type RNA was significantly more accessible to DMS modification, while the mutant RNA was highly inaccessible to DMS modification and the region preceding the stop codon remained unmodified (S6 Fig). These results indicate that ribosome pausing at the natural stop codon leads to destabilization of the downstream DS hairpin.

### The ring-like structure in the *speF* 5’ UTR affects RNA destabilization by arginine

**Fig 6:**
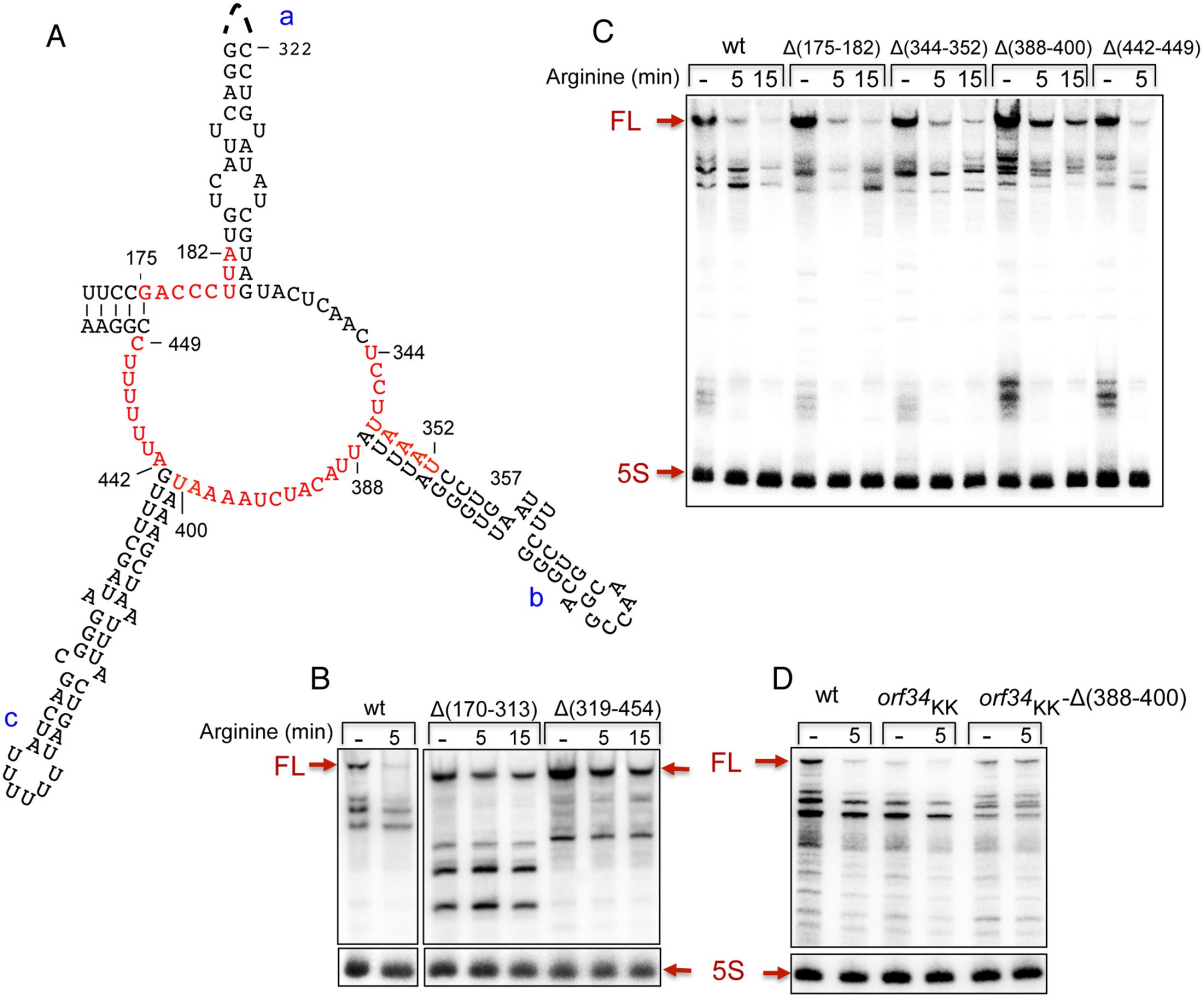
The ring-like structure affects RNA destabilization by arginine. (A) The ring structure as predicted by RNAfold is presented (see also Fig 2). The nucleotides deleted in C are marked in red. Long (B) and Short (C) deletions of ring sequences. Cultures with P*tac-orf34-speF* carrying deletions as indicated were grown and treated by arginine as described in Fig 3. The Northern blot was probed with end-labeled *orf34* (2411) and 5S rRNA (459) specific primers. (C) Deleting nucleotides 388–400 negates RNA destabilization by arginine. (D) Deleting nucleotides 388–400 negates the effect of *orf34*KK mutation. RNA analysis of wild type, P*tac-orf34*KK-*speF* and a double mutant P*tac-orf34*KK-Δ388-400-*speF* as indicated.

To examine the involvement of the putative ring-like structure and its sequence in RNA destabilization by arginine, we deleted two sequence elements of 143 (Δ170–313) and 135 (Δ319–454) nucleotides that comprise the 5‘ (proximal strand of hairpin a) and the 3’ (distal strand of hairpin a, and hairpins b and c) ends of the ring, respectively. RNA analysis of the two deletion mutants demonstrated that these regions play a role in mRNA destabilization in response to arginine (Fig 6A and 6B). To refine our deletion mapping, we also deleted the sequences constituting parts of the ring. RNA produced from the *orf34*-Δ388-400-*speF* construct in the presence of arginine displayed increased stability when compared to the wild type construct, indicating that deleting 12 nucleotides from position 388 to 400 affected the response to arginine and transcript stability (Fig 6C). Moreover, combing the Δ388–400 mutant that reduced RNA destabilization by arginine with the *orf34*KK mutant that exhibits no full-length transcript (Fig 3C) resulted in increased basal levels of the *orf34*KK-*speF* mRNA that was unaffected by arginine (Fig 6D). These results indicate that formation of the alternative ring-like structure facilitates *speF* mRNA decay.

## Discussion

In this study we showed that expression of *speF* in *Salmonella* is controlled at multiple levels through regulatory elements contained in the long leader sequence of the mRNA. A short open reading frame of 34 amino acids harboring a conserved domain functions at the heart of this regulation, required for *speF* production.

**Fig 7:**
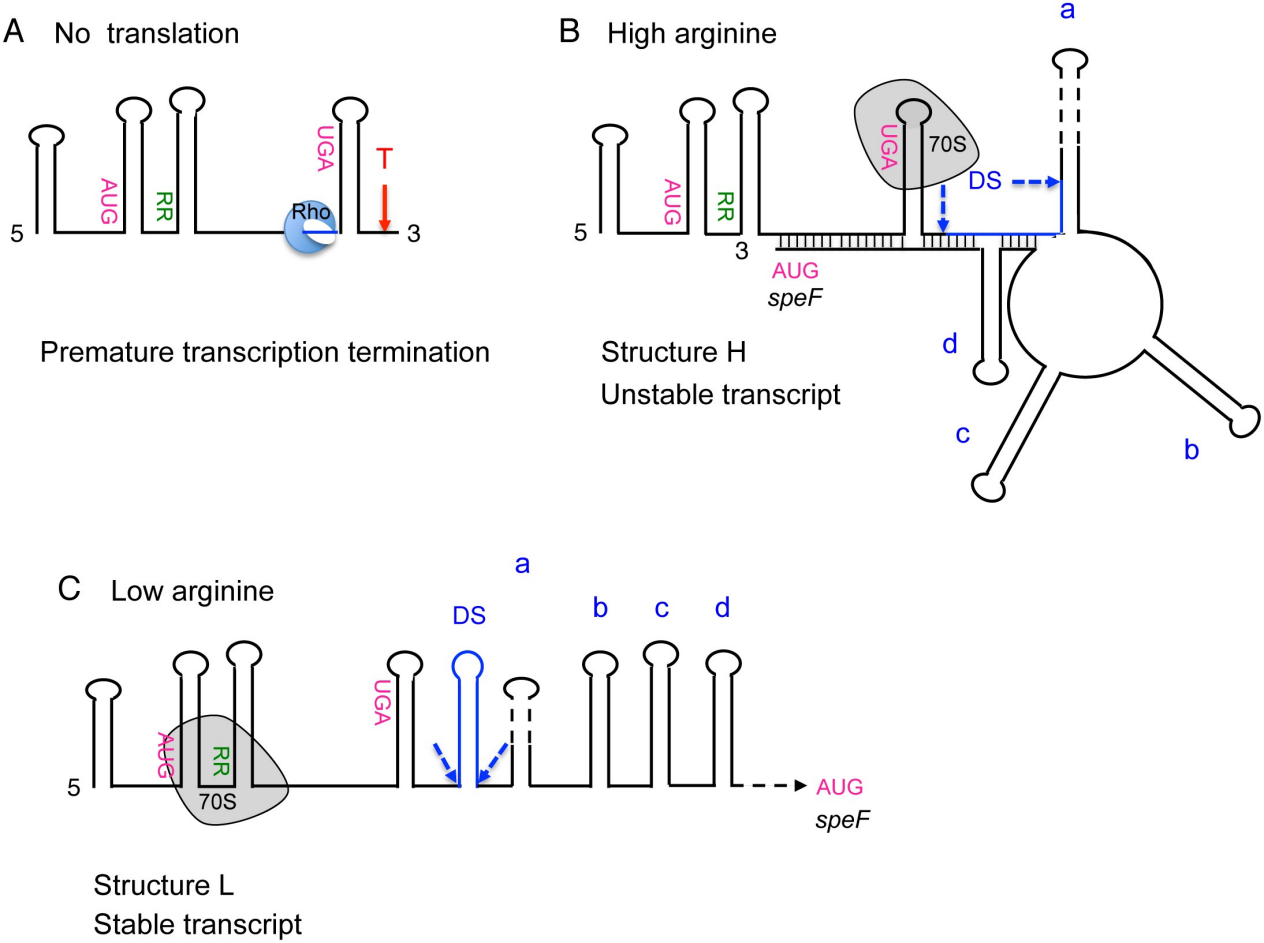
Post-transcriptional regulation of *speF* expression. (A) In the absence of *orf34* translation, or upon premature translation termination, the transcription termination factor Rho (in blue) binds *orf34* RNA, possibly at the CU-rich sequence that precedes the stop codon (blue line) leading to premature transcription termination of *orf34-speF* operon (T in red). (B) Under conditions of high arginine ribosomes translate *orf34* to its stop codon. Pausing at the stop codon prevents the formation of the ‘downstream of stop’ hairpin (DS; blue line) leading to the formation of the alternative structure H in which a ring-like structure is susceptible to degradation and the RBS of *speF* is inaccessible (C) Under low arginine ribosome attenuation at the consecutive arginine codons enables formation of hairpin DS (in blue), which in turn interferes with the formation of the ring-like structure. Consequently, the long transcript formed under low arginine (structure L) is stable and translationally active.

A key factor for *speF* regulation is the transcription termination factor Rho. Translation of *orf34* prevents Rho from transcription termination at the 5’ proximal region of the operon and unfolds an inhibitory structure that sequesters *speF* ribosome binding site. Rho contacts the RNA at Rho utilization (*rut*) sites, characterized by high pyrimidine residues with a preference for cytidine and relatively little secondary structure [28, 29]. The sequence of *speF* harbors a 12 nt long CU-rich domain opposite of the RBS of *speF* positioned at nucleotides 112 to 124. This sequence is similar to the CU-rich octamer identified in *Salmonella chiPQ* locus as the main *rut* site [14]. We propose that in the absence of translation or upon premature translational stop (UAA11 and UAA26), Rho binds the newly synthesized as yet unpaired CU-rich sequence opposite of the RBS, leading to premature transcription termination (Fig 7). Conversely, ribosomes translating *orf34* under conditions of high arginine, by reaching its stop codon sequester the putative Rho/anti-SD binding site preventing premature termination.

The consecutive arginine codons in the conserved domain of *orf34* provide an additional level of *speF* regulation. Under conditions of high arginine, uninterrupted translation of *orf34* results in the formation of a structure that is degraded by RNase G. Specifically, we propose that ribosomes pausing at the stop codon of *orf34* prevent formation of the ‘downstream of stop’ hairpin (DS), thus enabling the formation of an alternative conformation in which a ring-like RNA structure is formed. In this RNA structure the RBS of *speF* is inaccessible to the ribosome and becomes susceptible to degradation (Fig 7). Moving the *orf34* stop codon further downstream (*orf34*W35-*speF*) abolished regulation by arginine and the transcript produced remained stable in the presence of arginine (Fig 3D). Given that ribosomes translating beyond the natural position of *orf34* stop codon prevented regulation by arginine further confirms that ribosome pausing at this position is linked to the formation of an mRNA structure that is susceptible to decay. Furthermore, the structure probing data showing that the stop codon and the DS hairpins of wild type RNA were significantly more accessible to DMS modification than the RNA of *orf34*W35-*speF* mutant indicate that ribosomes pausing at the stop codon affect formation of the secondary structure downstream not by direct sequestration of the sequence required to form the DS hairpin but indirectly by affecting the rate of RNA polymerase progression and thus the dynamic of *speF* RNA folding. Numerous studies have shown that cooperation between the translating ribosomes and RNA polymerase influences the rate of transcription elongation and that direct binding between RNA polymerase and the first ribosome trailing the transcribing enzyme allows the polymerase to monitor translation rate [30–32]. We hypothesize that pausing of the translational machinery at the stop codon influences the dynamics of *speF* mRNA folding by affecting the rate of transcription elongation, hence leading to the formation of the alternative ring-like structure.

Unlike the *speF* transcript formed under high arginine conditions, the transcript formed under low arginine conditions is stable. Ribosome slow-down at the consecutive arginine codons in the absence of arginine enables the formation of hairpin DS, thus preventing formation of the ring-like RNA structure (Fig 7). Consequently, the transcript formed under low arginine is stable and translationally active. Deleting hairpin DS prevents formation of the unstable transcript, *i*.*e*. structure H, shifting the equilibrium towards structure L and the RNA remains stable in the presence of arginine. Overall, these results indicate that the rate of ribosome progression influences the dynamics of *speF* mRNA folding and thus the susceptibility of the transcript to ribonucleolytic degradation.

In addition to the long stable transcript produced under low arginine conditions, ribosome attenuation due to low arginine may also enable *rho*-dependent premature termination. Our results showing that compared to the full-length mRNA, the levels of the truncated transcripts increase by 12–13 fold in Δ*pnp* vs. wild type, indicate that indeed upon ribosome attenuation a large portion of the transcripts terminate. These transcripts undergo decay in wild type cells by 3’-end exoribonucleases and thus are undetectable.

For many riboswitches, e.g. the *thiM* riboswitch of *E*. *coli*, mRNA decay is triggered as a consequence of translation inhibition [33]. In contrast, in the *lysC* riboswitch, these two regulatory activities, translation initiation and mRNA decay, are independently controlled using the same conformational switch [33]. When bound to lysine the *lysC* riboswitch adopts a conformation that inhibits translation and promotes RNase E-mediated cleavage. In the absence of lysine, the transcript adopts an alternative conformation that allows translation initiation and sequesters the RNase E cleavage sites. Whereas for *thiM*, *lysC* and other riboswitches impaired translation is accompanied by mRNA decay, in *speF* regulation, uninterrupted translation of *orf34* results in mRNA decay. Translation of *orf34* under conditions of high arginine results in formation of a ring-like alternative RNA structure and our data indicate that this conformation is susceptible to degradation by RNase G. Deleting 12 nucleotides from position 388 to 400 of the ring affected the response to arginine and transcript stability. RNA produced from this mutant was more stable compared to wild type and basal RNA levels produced by the double mutant combing Δ388–400 with *orf34*KK were higher than those of *orf34*KK. Furthermore, mapping of the RNase G cleavage sites revealed a number of weak and strong sites at 10 min of exposure to arginine at which mRNA decay was more prominent. Notably, one strong site A392 resides within the sequence 388–400, further indicating that RNase G, by cleaving the ring-like sequence mediates mRNA decay of *speF* in the presence of arginine.

The *speF* gene encodes an inducible ornithine decarboxylase to produce putrescine, which can also be produced from arginine in an alternative pathway. The production of putrescine from arginine requires two steps, whereas ornithine produces putrescine in one-step and yet, arginine down-regulates *speF* expression. As the Km value of arginine decarboxylase is two-fold lower than that of ornithine decarboxylase [34, 35], it is reasonable that in the presence of arginine the alternative "shorter" pathway is turned off.

Unlike *speF* regulation by arginine in which a dramatic drop in *speF* mRNA levels was observed within a few minutes of exposure at exponential phase, induction by ornithine was detected upon a long exposure into stationary phase. Treating cultures at exponential phase with ornithine showed no increase in the levels of *speF* mRNA (S7A Fig). In contrast, a dramatic increase in the levels of this mRNA was detected in cultures grown to stationary phase in the presence of ornithine compared to untreated cultures. As *speF* promoter is unaffected by ornithine, we propose that the mRNA undergoes decay in untreated cultures growing to stationary phase, whereas in the presence of ornithine *speF* mRNA accumulates. Deleting the 5’-end region of the ring-like structure rendered the RNA more stable increasing its basal levels, further indicating that the ring-like structure is involved in *speF* decay and that the unstable mRNA of stationary phase wild type cells is stabilized in the presence of ornithine (S7B Fig). As induction by ornithine of Δ388–400 construct that is unresponsive to arginine and for the same reason to RNase G cleavage is similar to that of wild type, *speF* mRNA decay during growth to stationary phase is unrelated to RNase G mediated regulation in response to arginine. In addition, since northern analysis of stationary phase *speF* mRNA (P*tac*-*orf34-speF*) exhibits very low levels, whereas the levels of *speF-lacZ* fusions (P*speF-orf34-speF-lacZ*) are relatively high, it is conceivable that the β- galactosidase value of ~800 Miller units detected at 17 hours of growth is over represented due to the extended stability of the *lacZ* fusion transcript.

Based on our data we propose that consecutive arginine codons at positions 12 and 13, either rare or frequent, sense and transduce the signal of arginine availability. The 2-fold decrease in the basal levels of the *orf34*rRR-*speF*-*lacZ* transcription fusion carrying the consecutive identical CGACGA low usage arginine codons could be due to the phenomenon called 5’ ‘translational blockage’. Gao and collogues previously reported that upon ribosome attenuation due to consecutive identical low usage arginine codons positioned near the 5’ end of the message (at codon 13, as in *speF*) the ratio of free to bound mRNA was increased by 2-fold indicating that some mRNA was released from the ribosomes, undergoing decay [36]. Accordingly we compared the RNA levels of wild type and *orf34*rRR-*speF* at 17 hr. of growth, conditions under which the β- galactosidase activity assays were performed. The northern showed that the mRNA levels of the two strains were low and yet the levels of *orf34*rRR-*speF* were lower than that of wild type, which could explain the lower levels of LacZ activity (S8 Fig). Since the RNA levels of wild type and *orf34*rRR-*speF* mutant at exponential phase were comparable (Fig 3E and S8 Fig), we suspect that *orf34*rRR-*speF* transcript becomes more susceptible to decay as the activity of ribosome attenuation at the consecutive identical rare arginine codons overlaps with stationary phase.

The elements for the arginine-dependent control reside within the conserved core of ORF34 that is widespread among γ-proteobacteria, many of which are pathogenic, and the homologues of ORF34 typically precede the inducible version of ornithine decarboxylase. We find that addition of ornithine or arginine supports the growth of wild type *Salmonella* as compared to Δ*orf34-speF* mutant (S9 Fig) indicating that this regulatory *cis*-element and the availability of arginine and/or ornithine play a role in bacterial metabolism under normal growth conditions. Whether this conserved regulatory *cis*-element plays a role in bacterial virulence during infection or the rapid switch between the two alternative biosynthetic pathways is used as a checkpoint affecting both the host and its assailant is an intriguing thought for future studies.

Polyamines are widely distributed in nature, they bind nucleic acids and proteins and although their exact mechanism of action is not clear, their effect on fundamental cellular functions is well documented [22]. Accordingly, the intracellular concentrations of polyamines are tightly controlled and so are the enzymes involved in polyamine production. The canonical biosynthesis pathway of polyamines is conserved and begins with ornithine decarboxylase that forms putrescine. Not surprisingly, the intracellular levels of ornithine decarboxylase are strictly regulated at multiple levels by various mechanisms. Our results show that despite the variations in the mechanistic details, the regulatory elements of ornithine decarboxylase *i*.*e*., the upstream ORF and the structured 5’UTR are evolutionary conserved from bacteria to mammals.

## Materials and methods

### Bacterial growth conditions

*Salmonella* Typhimurium SL1344 cells were grown at 37°C (200 rpm) in Luria-Bertani (LB) broth (pH 6.8) or in E-minimal medium (Vogel-Bonner medium). The salt composition of E-min is MgSO4(H2O) (0.2 mg/ml), citric acid monohydrate (2 mg/ml), K2HPO4 (10 mg/ml), NaNH4PO4 (3.5 mg/ml). Histidine (100 μg/ml), glucose (0.4%) and vitamin B1 (2 μg/ml) were added after autoclave. Where indicated, L-ornithine (25, 50 or 100 μg/ml) or arginine (100 μg/ml) was added. Ampicillin (100 μg/ml) and kanamycin (40 μg/ml) were added where appropriate.

### Plasmid construction

To construct Ptac-*orf34*-*speF*, a fragment of 616 nt of *orf34-speF*, spanning from the transcription start site to nucleotide +101 with respect to the *speF* AUG was PCR amplified from *S*. *typhimurium* SL1344 chromosomal DNA using primers 1788 and 1790 and cloned into the EcoRI and HindIII restriction sites of pRI. To construct P*speF-orf34-speF-lacZ* transcriptional and translational fusions, a fragment of 817 nt (from nucleotide 201 upstream of *speF* transcription start site to nucleotide +101 with respect to the *speF* AUG) carrying the promoter of *speF*, *orf34* and part of *speF* was PCR amplified from *S*. *typhimurium* SL1344 chromosomal DNA using primers 1636 and 1637 and cloned into the EcoRI and BamHI restriction sites of pRS551 and pRS552, respectively. To construct P*speF*-*orf34*-*lacZ* transcriptional and translational fusions, a fragment of 334 nucleotides (from nucleotide 201 upstream of *speF* transcription start site to nucleotide +102 with respect to the *orf34* AUG) carrying the promoter of *speF* and *orf34* was PCR amplified from *S*. *typhimurium* SL1344 chromosomal DNA using primers 1636 and 1639 and cloned into the EcoRI and BamHI restriction sites of pRS551, pRS552 respectively. To fuse the promoter of *speF* operon to *lacZ* (P*speF*-*lacZ*) a fragment of 215 nucleotides (from nucleotide 201 upstream of *speF* transcription start site to nucleotide 14 down stream of it) was PCR amplified from *S*. *typhimurium* SL1344 chromosomal DNA using primers 1636 and 1898 and cloned into the EcoRI and BamHI restriction sites of pRS551. To construct PL*tetO-1-orf34* (p15A origin), the *orf34* sequence was PCR amplified from +5 of the transcription start site up to 9 nt downstream of the stop codon using primers 2368 (KpnI) and 2369 (phosphorylated). The two PCR fragments (*orf34* and pZA31) were then ligated.

### Strain construction

*speF* deletion mutant in *S*. *typhimurium* SL1344 strain was constructed based on the gene disruption method [37]. Chloramphenicol cassette was amplified by PCR using primers 1831 and 1832 containing sequences homologous to *orf34*-*speF*. The DNA generated was transformed into LB5010 cells carrying pKD46 plasmid and chloramphenicol resistant colonies were selected. *speF* gene disruption was examined by PCR using flanking primers, 1833 and 1834. To insert the *lacZ* fusions as single copies in the *hisG* gene sequence in the chromosome, DNA fragments including kanamycin and *speF-lacZ* fusions were PCR amplified using 1514 and 1515 primers. Ampicillin sensitive and kanamycin resistant cells were selected. The insertions into *hisG* gene were examined by PCR using flanking primers, 1517 and 1518. The chromosomal fusions and the deletion mutant were moved into *S*. *typhimurium* SL1344 cells by P22 transduction.

### Site directed mutagenesis

Mutants Ps*peF*-*orf34*AAA-*speF*, P*speF*-*orf34*UAA16_-_*speF*, P*speF*-*orf34*UAA26_-_*speF*, P*speF*-*orf34*rRR-*speF*, P*speF*-*orf34*fRR_-_*speF*, P*speF*-*orf34*KK_-_*speF*, P*speF*-*orf34*CA116,117GT-*speF*, P*speF*-*orf34*TG510,511AC-*speF* were generated by whole plasmid PCR using pGEM4 carrying P*speF*-*orf34*AUG-*speF* and two tail-to-tail divergent primers of which, one or both, carried the desired mutation. The PCR product was subjected to blunt end ligation and transformed into MC4100. DNA fragments carrying the mutations were digested from pGEM4 using EcoRI and BamHI and cloned into pRS551 and pRS552. The deletion mutants were generated using divergent primers spaced by the desired deletion.

### β-galactosidase assays

Overnight cultures were diluted 1/100 in 7 ml LB medium supplemented with ampicillin and kanamycin in non-aerated 50 ml tubes and grown to OD600 of 0.9. Where indicated, bicyclomycin (BCM 20 μg /ml) was added at OD600 of 0.2 for 1 hour. To examine regulation by arginine or ornithine, colonies were inoculated in 5ml E-minimal medium in non-aerated 50 ml tubes for 17 hours. Each colony was inoculated evenly in arginine plus and minus tubes. Arginine or ornithine (100 μg /ml) was added where indicated from the beginning of growth.

### RNA extraction

Overnight cultures of *S*. *typhimurium* SL1344 carrying P*tac*-*orf34*-*speF* plasmids were diluted 1/100 in 20 ml LB medium supplemented with ampicillin in 125ml flasks and grown to OD600 of 0.2. Where indicated, BCM (20 μg/ml) was added at OD600 of 0.2 for 1 hour. To examine regulation by arginine or ornithine, overnight cultures were diluted 1/50 in 20 ml E-minimal medium in 125 ml flasks and grown to OD600 of 0.2 at which arginine or ornithine (100 μg/ml) was added for 5 or 15 minutes. For ornithine induction at stationary phase, cultures were grown in E-minimal medium in the presence of ornithine (25 or 50 μg/ml). Total RNA was purified using TRI reagent (Sigma) according to the manufacturer’s protocol.

### Northern analysis

RNA samples (10 μg) were denatured for 10 min at 70°C in 98% formamide loading buffer, separated on 8M urea-6% polyacrylamide gels and transferred to Zeta Probe GT membranes (Bio-Rad Laboratories) by electroblotting. To detect *orf34*-*speF* mRNA, the membranes were hybridized in modified CHURCH buffer using [32P]-end labeled 1614 primer ([38]. Since 1614 primer overlaps the *orf34* consecutive arginine codons, *orf34* arginine codon mutants were detected using 2411 primer. 5S rRNA was used as a loading control using primer 459. After 2 hours at 45°C, the membranes were washed for 20 min. in 3XSSC at 45°C.

### 3' RACE

The site of premature termination in *orf34*AAA mutant was determined by 3'-RACE as described before [39] using 10 μg of RNA extracted from *S*. *typhimurium* SL1344 culture carrying Ptac-*orf34*AAA-*speF* plasmid. RNA adapter (1579) and primer 2211 were used for reverse transcription reaction. 1 μl of the cDNA product was PCR amplified using primers 2212 and 2213 and cloned into the AatII and HindIII restriction sites of pBR plasmid. The ligation product of pBR with *speF*-E1 insert was electro-transformed into MC4100 cells. Plasmid was purified and sequenced to detect the exact position of premature termination of *speF*.

## Supporting information

S1 Fig An enrooted phylogenetic tree of representative DUF2618 containing peptides in γ-proteobacteria. (A) Sequence studied in this work is marked in purple. Representative proteins were chosen from NCBI collection of DUF2618 containing proteins. Additional protein sequences were obtained from full genomes found by tBlastn using *orf34* coding sequence as query. Multiple sequence alignments were created using ClustalX v.2.1 [1] Maximum-likelihood phylogenetic trees were constructed using the phylogeny.fr pipeline [2], including the PhyML v.3.0 [3] and the WAG substitution model for amino acids [4]. One hundred bootstrap replicates were performed for each analysis. Branches having branch support value smaller than 85% confidence were collapsed. (B) Multiple sequence alignment of DUF2618 containing representatives by ClustalX v 2.1 (C) Protein logo of DUF2618 containing proteins. Proteins containing DUF2618 (PF10940) alignment was generated from NCBI (43 non-redundant proteins) using Pfam (http://pfam.xfam.org/) [5]. Alignment was used to generate a consensus protein logo (https://rth.dk/resources/plogo/) [6],[7]. Amino acids are marked by one letter, the polarity of the amino acids side chain marked by color; positive (blue), negative (red), uncharged (green) or hydrophobic (black). The numbers represent the corresponding position in ORF34.
(PDF)

S2 Fig *speF* mRNA decay is significantly slow in *rng* mutant compared to wild type. (A) Cultures of wild type and *rng* mutant carrying P*tac-orf34-speF* were grown in E-minimal media to OD600 of 0.2 and then treated with arginine (100 μg /ml) for the indicated time. Northern blot of RNA samples (10 μg) separated using 6% urea-polyacrylamide gels. The membranes were probed with end-labeled *orf34* (2411) and 5S rRNA (459) specific primers. 5S RNA serves as a loading control. Full-length (FL) is indicated in red. Relative intensity of the full-length RNA as quantified using ImageQuant TL 1D v8.1 is shown (R.I of FL). (B) Graphs of relative band intensity of the data presented in A.
(PDF)

S3 Fig Primer extension of RNA samples of wild type and *rng* shown in S2 Fig using primers 1825 left panel 2429 right panel. (PDF)

S4 Fig Mapping of RNAse G cleavage sites detected by primer extension (see S3). Arrowheads and arrows indicate weak and strong cleavage sites respectively. Not all site are shown.
(PDF)

S5 Fig Cultures of wild type, RNase G mutant (Δ*rng*) and RNase E temperature sensitive mutant (*rne*-6) carrying P*tac-orf34-speF* were grown in E-minimal media to OD600 of 0.2 at 30°C and then transferred to 42°C for 30 min. prior to the arginine treatment (100 μg /ml). The membranes were probed with end-labeled *orf34* (1614) and 5S rRNA (459) specific primers.
(PDF)

S6 Fig Ribosome attenuation at the stop codon destabilizes the nearby structures. *In vivo* structure probing. Cultures of RNase G mutant (Δ*rng*) carrying P*tac-orf34-speF* and P*tac-orf34*W35-*speF* in which the stop codon was changed to a Trp codon were grown in E-minimal media to OD600 of 0.2 and then treated with arginine (100 μg /ml) for 5 min and DMS (1/700) for 2.5 min (overlapping). Primer extension of 2 μg (no DMS) and 8 μg (plus DMS) of total RNA using 1789 primer. The data probing obtained with wild type RNA are displayed on the structure on the left. Red and blue asterisks indicate strong and weak modification sites. Data obtained with *orf34*W35 mutant are displayed on the structure on the right. Purple and grey asterisks indicate weak and faint modification sites. Note that wild type RNA is much more accessible to DMS modification including the region upstream of the stop codon, while the mutant RNA is much less accessible to DMS modification and the region upstream of the stop codon remains unmodified.
(PDF)

S7 Fig *speF* mRNA induction by ornithine. (A) Cultures carrying P*tac-orf34-speF* were grown in E-minimal media to OD600 of 0.2 and then exposed to ornithine (100 μg /ml) for the indicated time or left untreated (-). (B) Cultures carrying P*tac-orf34-speF* wild type, Δ(170–313) and Δ(388–400) were grown in E-minimal media to OD600 of 2.0 in the presence of ornithine (25 or 50 μg /ml) or left untreated (-). Northern blot of RNA samples (10 μg) separated using 6% urea-polyacrylamide gels. The membranes were probed with end-labeled *orf34* (1614) and 5S rRNA (459) specific primers. 5S RNA serves as a loading control. Full-length (FL) and 5S are indicated in red.
(PDF)

S8 Fig *speF* mRNA levels of wild type and *orf34rRR* at log and stationary phase. Cultures carrying wild type P*tac-orf34-speF* and P*tac-orf34rRR-speF* were grown in E-minimal media to OD600 of 0.2 (Log) or for 17 hours to stationary phase (Stationary). Northern blot of RNA samples (left panel 10 μg and right panel 8 μg) separated using 6% urea-polyacrylamide gels. The membranes were probed with end-labeled *orf34* (2411) and 5S rRNA (459) specific primers. Left panel was exposed for 2 hours while the right panel was exposed for 8.5 hours. 5S RNA serves as a loading control. Full-length (FL) and 5S are indicated in red.
(PDF)

S9 Fig The addition of ornithine or arginine supports the growth of wild type compared to Δ*speF*. Cultures of wild type and Δ*speF* were grown in E-minimal medium supplemented with ornithine (100 μg /ml) or arginine (100 μg /ml) as indicated or were left untreated. Note that in the absence of ornithine or arginine the growth rate of the mutant is similar to that of the wild type, whereas upon addition of ornithine or arginine wild type cells grow better than Δ*speF*. Average of two biological experiments ± standard deviation.
(PDF)

S1 Table Results are displayed as mean of 2 colonies ± standard deviation. Cultures were grown for 17 hours from a single colony in 5 ml (50 ml tubes) of E-Minimal supplemented with arginine (100 μg /ml). PL*tetO* and PL*tetO*-*orf34* are P15A origin.
(DOCX)

S2 Table Strains used in this study. (DOCX)

S3 Table ^a^Primers used for fragment amplification. ^b^Primers used to generate mutations.
(DOCX)

S4 Table (A) ^a^Plus (+) and minus (-) strands are indicated. (B) ^a^Plus (+) and minus (-) strands are indicated. (C) ^a^Plus (+) and minus (-) strands are indicated. ^b^All mutant constructs are based on 616 nt fragment (1636–1637) consisting of P*speF-orf34-speF'-lacZ*. ^c^Positions of the mutations or deletions are relative to the transcription start site of *speF* operon transcript.
(DOCX)

S1 Text Additional references. (DOCX)
