# Supplementary figures and images for: mRNA dynamics and alternative conformations adopted under low and high arginine concentrations control polyamine biosynthesis in Salmonella
Source: PLoS Genet. 2019 Feb 11;15(2):e1007646. doi: 10.1371/journal.pgen.1007646 (PMC6386406; doi:10.1371/journal.pgen.1007646)

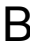[illegible]

Supplement: S1 Fig — (A) Sequence studied in this work is marked in purple. Representative proteins were chosen from NCBI collection of DUF2618 containing proteins. Additional protein sequences were obtained from full genomes found by tBlastn using orf34 coding sequence as query. Multiple sequence alignments were created using ClustalX v.2.1 [1] Maximum-likelihood phylogenetic trees were constructed using the phylogeny.fr pipeline [2], including the PhyML v.3.0 [3] and the WAG substitution model for amino acids [4]. One hundred bootstrap replicates were performed for each analysis. Branches having branch support value smaller than 85% confidence were collapsed. (B) Multiple sequence alignment of DUF2618 containing representatives by ClustalX v 2.1 (C) Protein logo of DUF2618 containing proteins. Proteins containing DUF2618 (PF10940) alignment was generated from NCBI (43 non-redundant proteins) using Pfam (http://pfam.xfam.org/) [5]. Alignment was used to generate a consensus protein logo (https://rth.dk/resources/plogo/) [6],[7]. Amino acids are marked by one letter, the polarity of the amino acids side chain marked by color; positive (blue), negative (red), uncharged (green) or hydrophobic (black). The numbers represent the corresponding position in ORF34. (PDF) [file pgen.1007646.s001.pdf]

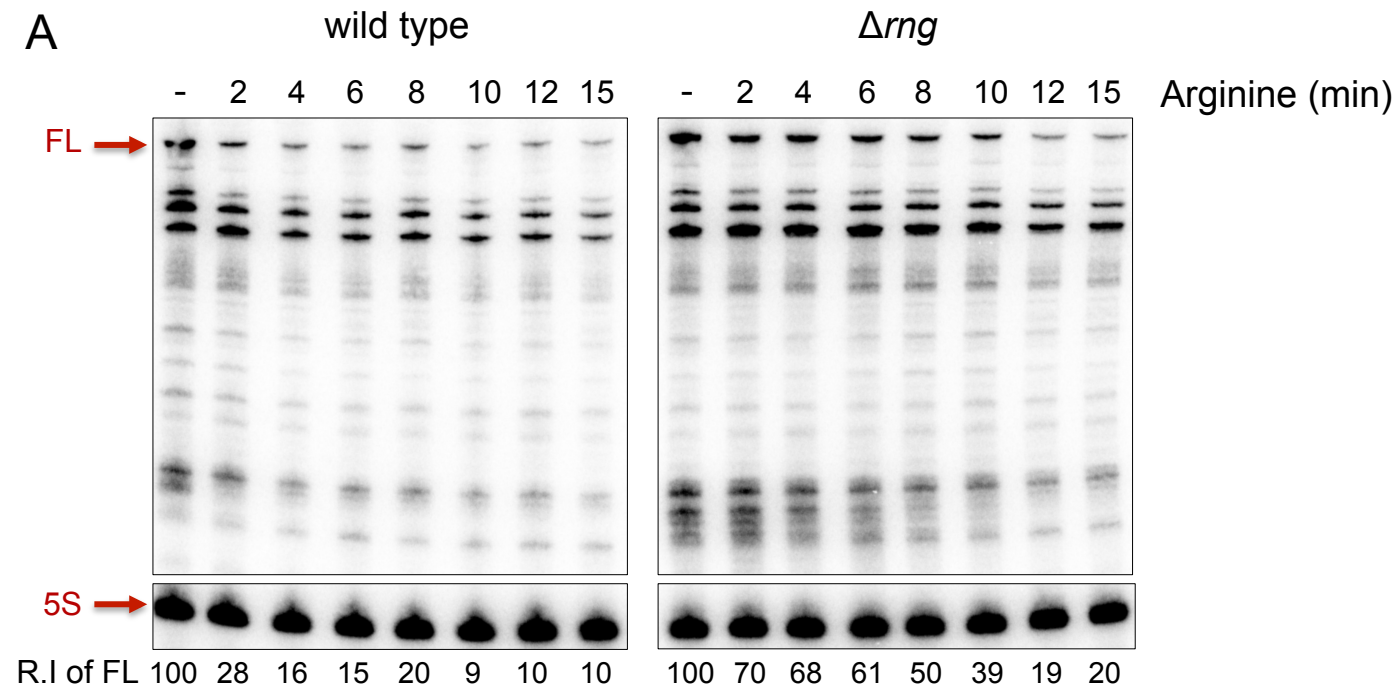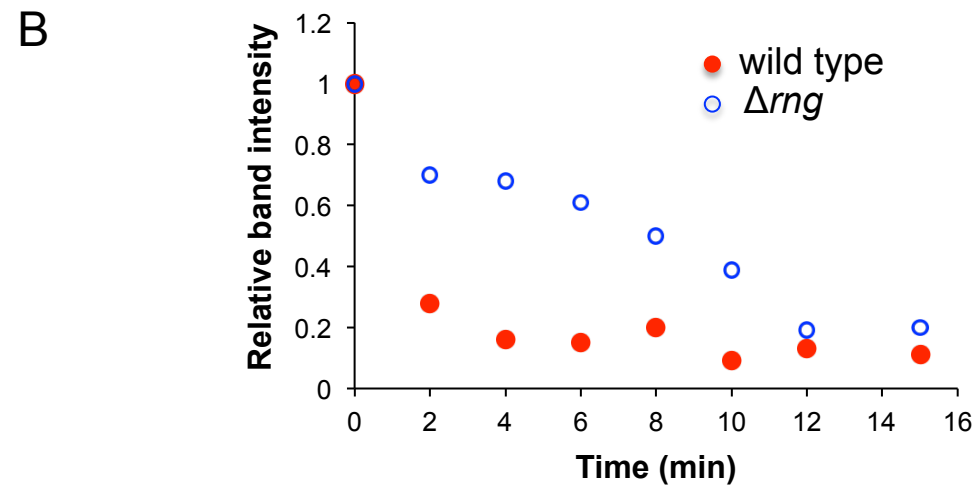

Supplement: S2 Fig — (A) Cultures of wild type and rng mutant carrying Ptac-orf34-speF were grown in E-minimal media to OD600 of 0.2 and then treated with arginine (100 μg /ml) for the indicated time. Northern blot of RNA samples (10 μg) separated using 6% urea-polyacrylamide gels. The membranes were probed with end-labeled orf34 (2411) and 5S rRNA (459) specific primers. 5S RNA serves as a loading control. Full-length (FL) is indicated in red. Relative intensity of the full-length RNA as quantified using ImageQuant TL 1D v8.1 is shown (R.I of FL). (B) Graphs of relative band intensity of the data presented in A. (PDF) [file pgen.1007646.s002.pdf]

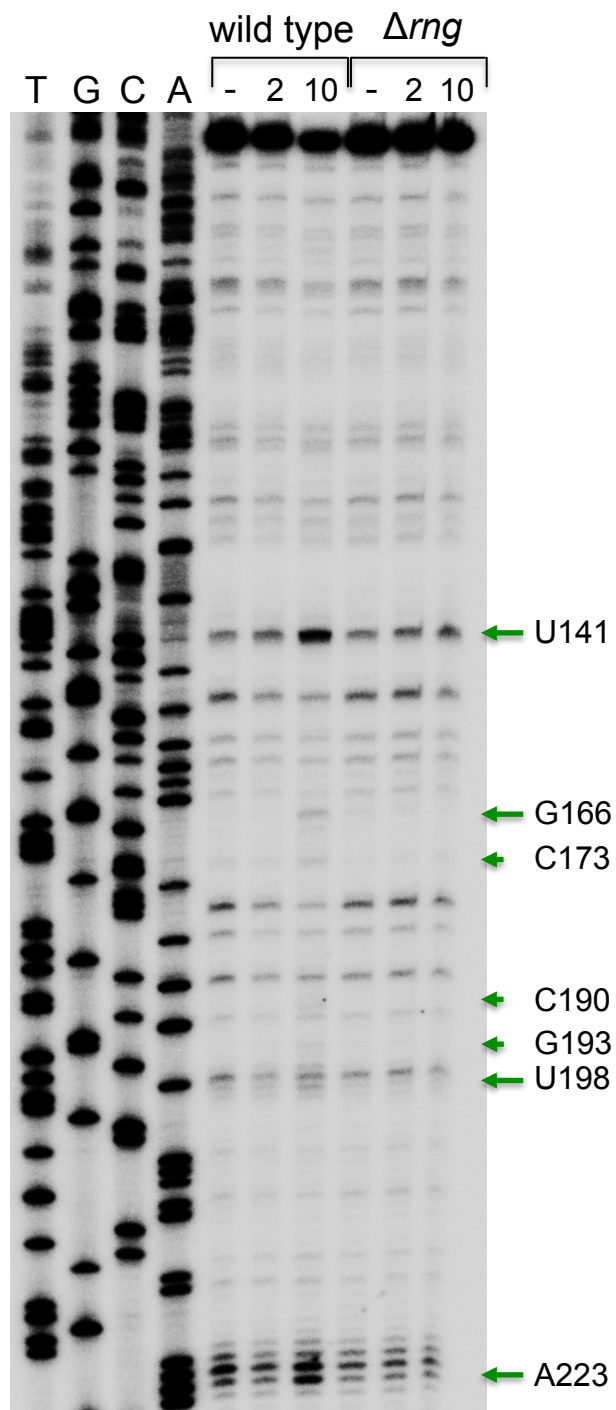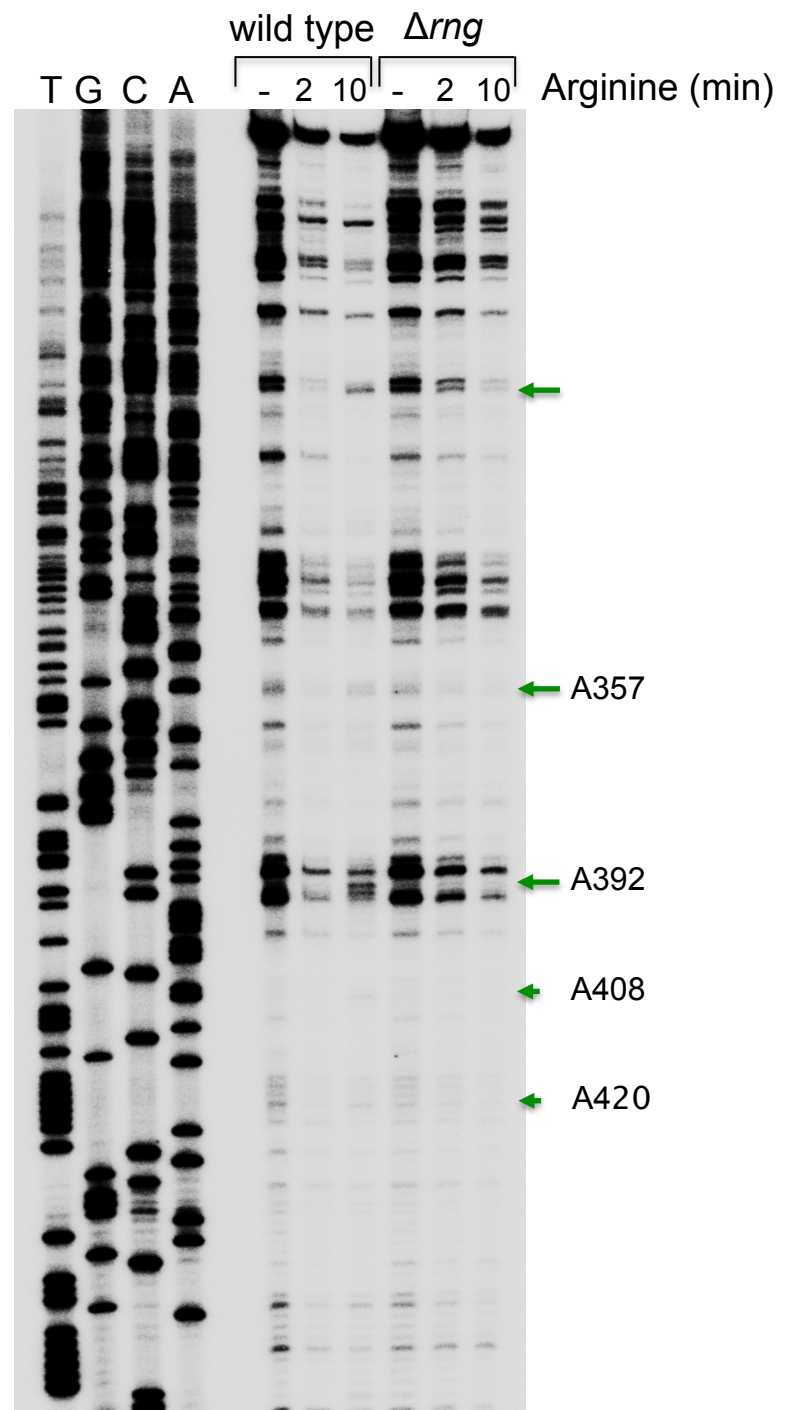

Supplement: S3 Fig — (PDF) [file pgen.1007646.s003.pdf]

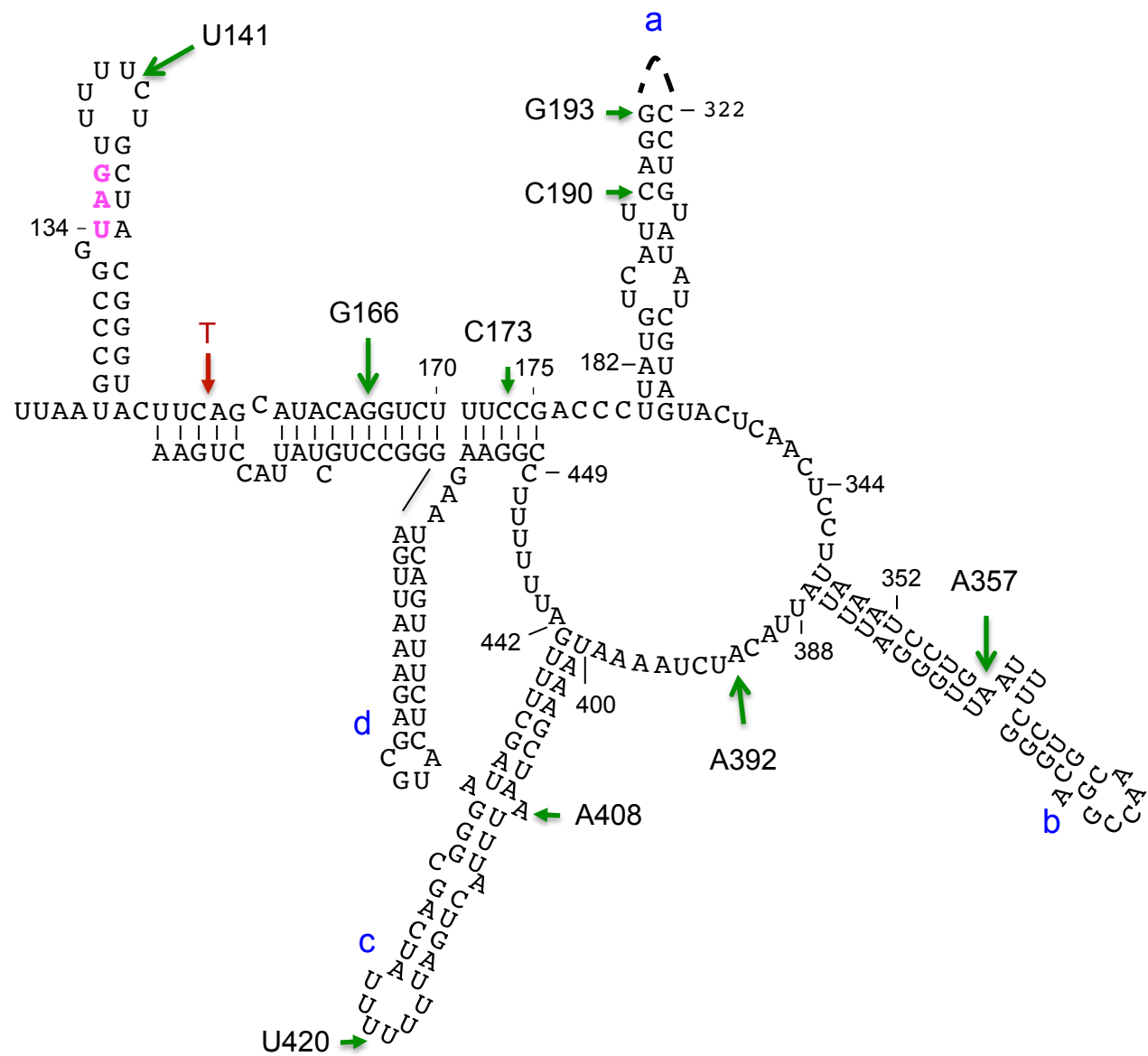

Supplement: S4 Fig — Arrowheads and arrows indicate weak and strong cleavage sites respectively. Not all site are shown. (PDF) [file pgen.1007646.s004.pdf]

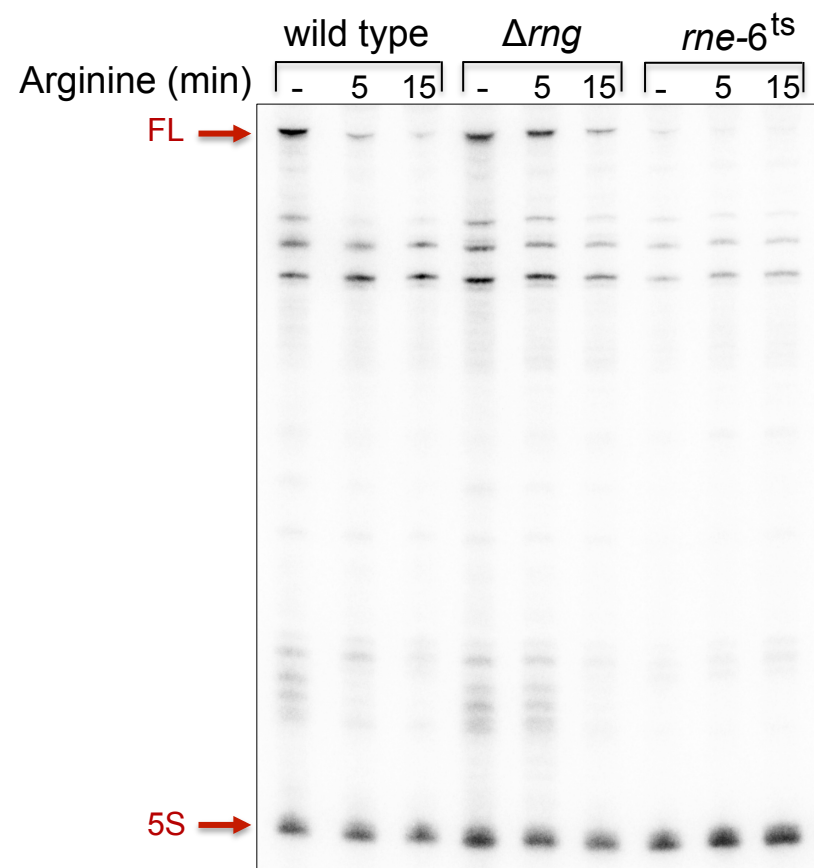

Supplement: S5 Fig — prior to the arginine treatment (100 μg /ml). The membranes were probed with end-labeled orf34 (1614) and 5S rRNA (459) specific primers. (PDF) [file pgen.1007646.s005.pdf]

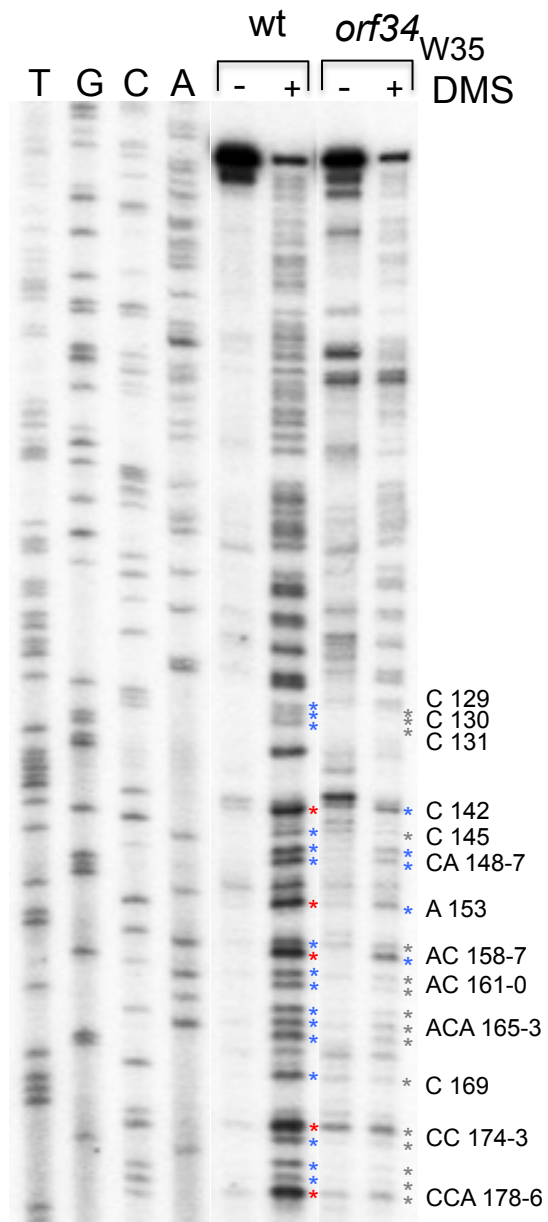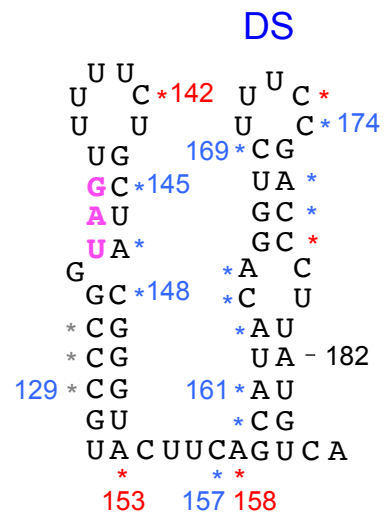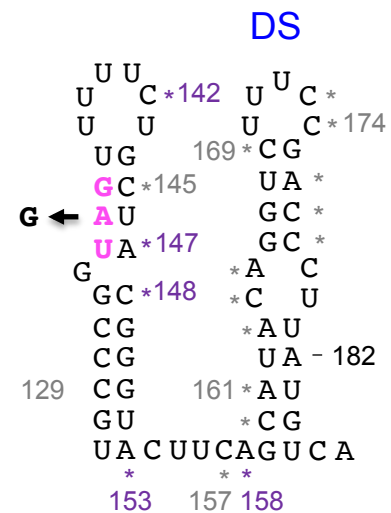

Supplement: S6 Fig — In vivo structure probing. Cultures of RNase G mutant (Δrng) carrying Ptac-orf34-speF and Ptac-orf34W35-speF in which the stop codon was changed to a Trp codon were grown in E-minimal media to OD600 of 0.2 and then treated with arginine (100 μg /ml) for 5 min and DMS (1/700) for 2.5 min (overlapping). Primer extension of 2 μg (no DMS) and 8 μg (plus DMS) of total RNA using 1789 primer. The data probing obtained with wild type RNA are displayed on the structure on the left. Red and blue asterisks indicate strong and weak modification sites. Data obtained with orf34W35 mutant are displayed on the structure on the right. Purple and grey asterisks indicate weak and faint modification sites. Note that wild type RNA is much more accessible to DMS modification including the region upstream of the stop codon, while the mutant RNA is much less accessible to DMS modification and the region upstream of the stop codon remains unmodified. (PDF) [file pgen.1007646.s006.pdf]

A

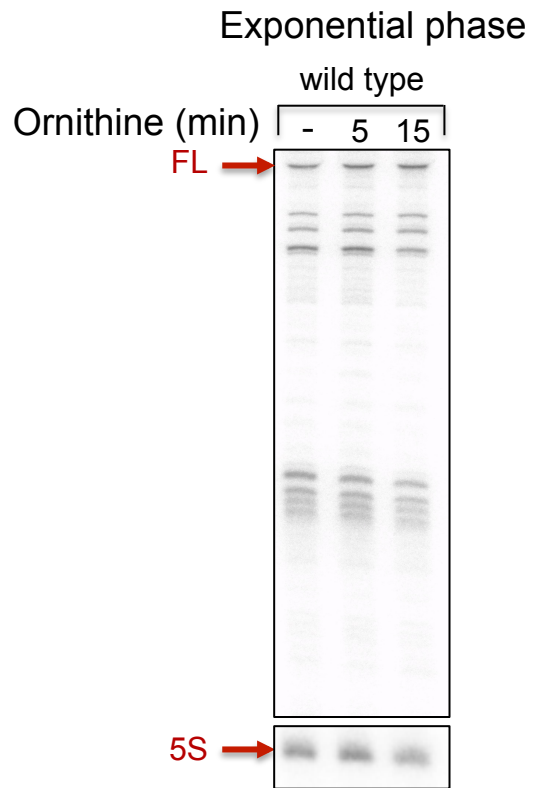

B

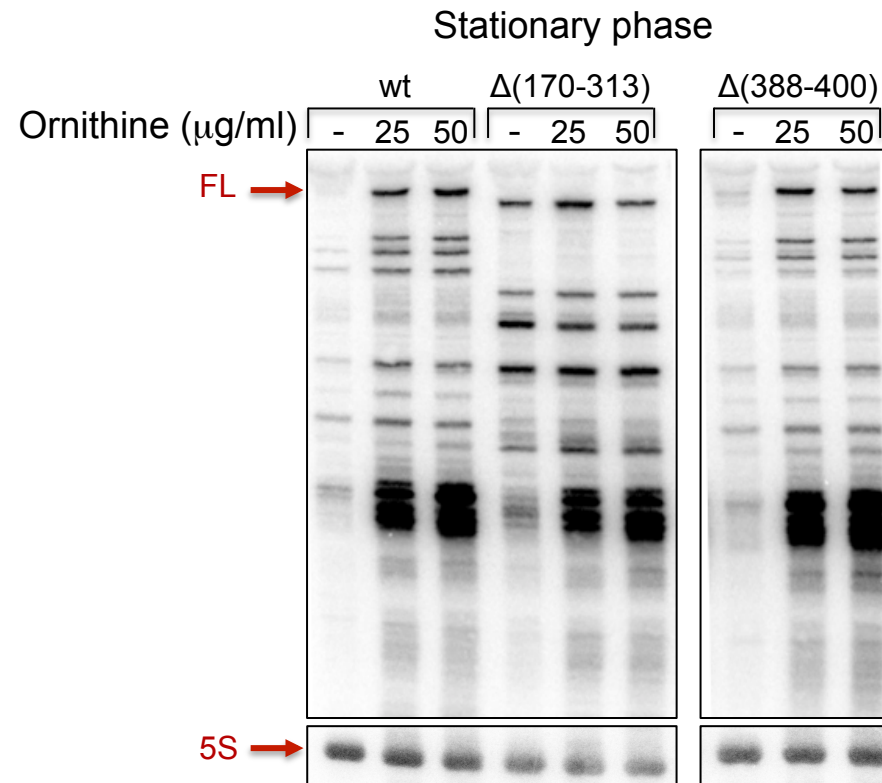

Supplement: S7 Fig — (A) Cultures carrying Ptac-orf34-speF were grown in E-minimal media to OD600 of 0.2 and then exposed to ornithine (100 μg /ml) for the indicated time or left untreated (-). (B) Cultures carrying Ptac-orf34-speF wild type, Δ(170–313) and Δ(388–400) were grown in E-minimal media to OD600 of 2.0 in the presence of ornithine (25 or 50 μg /ml) or left untreated (-). Northern blot of RNA samples (10 μg) separated using 6% urea-polyacrylamide gels. The membranes were probed with end-labeled orf34 (1614) and 5S rRNA (459) specific primers. 5S RNA serves as a loading control. Full-length (FL) and 5S are indicated in red. (PDF) [file pgen.1007646.s007.pdf]

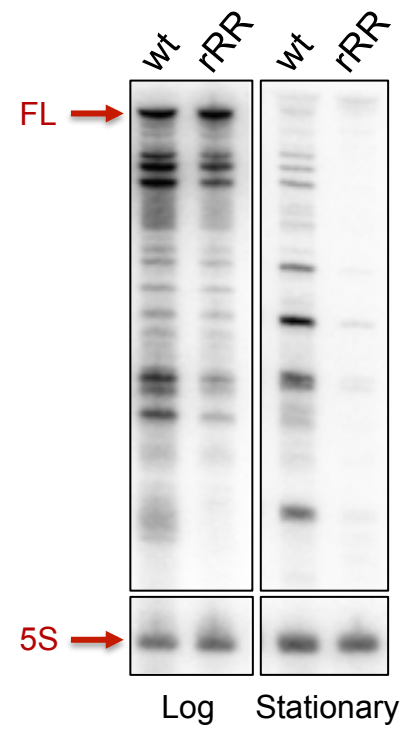

Supplement: S8 Fig — Cultures carrying wild type Ptac-orf34-speF and Ptac-orf34rRR-speF were grown in E-minimal media to OD600 of 0.2 (Log) or for 17 hours to stationary phase (Stationary). Northern blot of RNA samples (left panel 10 μg and right panel 8 μg) separated using 6% urea-polyacrylamide gels. The membranes were probed with end-labeled orf34 (2411) and 5S rRNA (459) specific primers. Left panel was exposed for 2 hours while the right panel was exposed for 8.5 hours. 5S RNA serves as a loading control. Full-length (FL) and 5S are indicated in red. (PDF) [file pgen.1007646.s008.pdf]

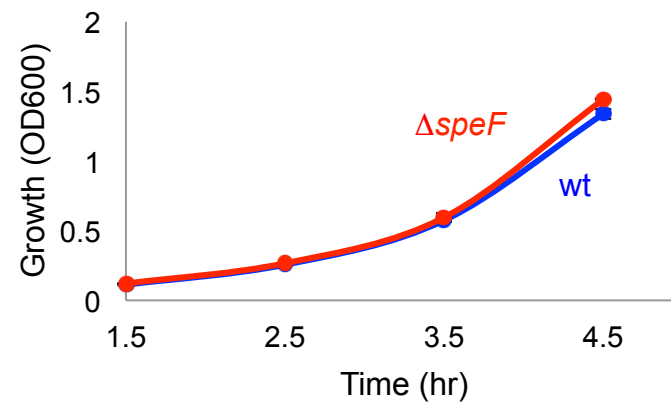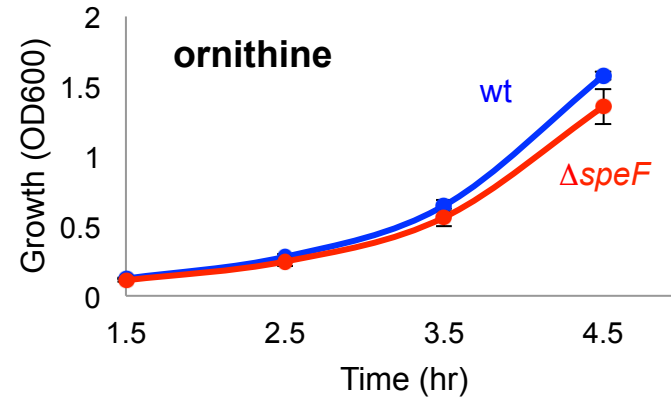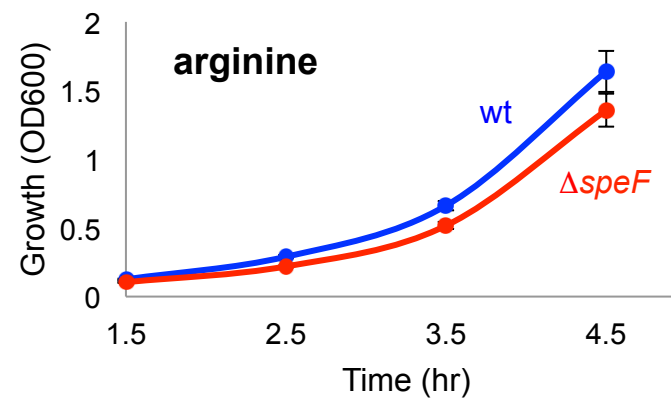

Supplement: S9 Fig — Cultures of wild type and ΔspeF were grown in E-minimal medium supplemented with ornithine (100 μg /ml) or arginine (100 μg /ml) as indicated or were left untreated. Note that in the absence of ornithine or arginine the growth rate of the mutant is similar to that of the wild type, whereas upon addition of ornithine or arginine wild type cells grow better than ΔspeF. Average of two biological experiments ± standard deviation. (PDF) [file pgen.1007646.s009.pdf]
