# Supplementary material for: mRNA dynamics and alternative conformations adopted under low and high arginine concentrations control polyamine biosynthesis in Salmonella
Source: PLoS Genet. 2019 Feb 11;15(2):e1007646. doi: 10.1371/journal.pgen.1007646 (PMC6386406; doi:10.1371/journal.pgen.1007646)
Supplement: S1 Table — Cultures were grown for 17 hours from a single colony in 5 ml (50 ml tubes) of E-Minimal supplemented with arginine (100 μg /ml). PLtetO and PLtetO-orf34 are P15A origin. (DOCX) [file pgen.1007646.s010.docx]

**S1 Table**. In *trans* expression of *orf34* (β-galactosidase activity)

| Genotype | Transcription fusion | |  | Translation fusion | |  |
| --- | --- | --- | --- | --- | --- | --- |
|  | - Arginine | + Arginine |  | - Arginine | + Arginine |  |
| 1. P-Δ(*orf34*)*-speF-lacZ*/ PL*tetO* | 683 ± 49 | 647 ± 65 |  | 276 ± 10 | 278 ± 26 |  |
| 2. P-Δ(*orf34*)*-speF-lacZ*/ PL*tetO*-*orf34* | 652 ± 47 | 515 ± 29 |  | 190 ± 33 | 155 ± 8 |  |
| 3. P-*orf34*AAA*-speF-lacZ*/ PL*tetO* | 265 ± 11 | 241 ± 38 |  | 2 ± 1 | 1 ± 1 |  |
| 4. P-*orf34*AAA*-speF-lacZ*/ PL*tetO*-*orf34* | 224 ± 7 | 226 ± 5 |  | 1 ± 1 | 1 ± 1 |  |
