# Supplementary material for: mRNA dynamics and alternative conformations adopted under low and high arginine concentrations control polyamine biosynthesis in Salmonella
Source: PLoS Genet. 2019 Feb 11;15(2):e1007646. doi: 10.1371/journal.pgen.1007646 (PMC6386406; doi:10.1371/journal.pgen.1007646)
Supplement: S2 Table — (DOCX) [file pgen.1007646.s011.docx]

**S2 Table. Strains**

| MC4100 | *E. coli F- araD139 Δ(argF-lac)U169 rpsL150 relA1 flbB5301 deoC1 ptsF25 rbsR* | Lab collection | A-2 |
| --- | --- | --- | --- |
| SL1344 | *S. typhimurium*, *hisG*46 | Lab collection | A-112 |
| SL1344 | Δ*orf34*-*speF::frt* | This study | A-765 |
| SL1344 | ∆*hisG*46::λRS551 (*kan*: P*speF*-*orf34'*-lacZ) | This study | D-672 |
| SL1344 | ∆*hisG*46::λRS552 (*kan*: P*speF*-*orf34'*-'lacZ) | This study | D-674 |
| SL1344 | ∆*hisG*46::λRS551 (*kan*: P*speF*-*orf34*AAA*-lacZ*) | This study | D-720 |
| SL1344 | ∆*hisG*46::λRS552 (*kan*: P*speF*-*orf34*AAA*-'lacZ*) | This study | D-722 |
| SL1344 | ∆*hisG*46::λRS551 (*kan*: P*speF-lacZ*) | This study | D-778 |
| SL1344 | ∆*cafA::cat* (RNase G deletion mutant) | JVS-00963 | A-668 |
| LT2 | *rne*-6ts:*tet* (50 % linkage; 37˚C) | [8] | A-792 |
| SL1344 | *rne*-6ts:*tet* (50 % linkage; 37˚C) | P22 of A792 | A-796 |
| SL1344 | ∆*pnp*::*cat* | P22 from JVS-00927 (A-672) | A-767 |
| LB5010 | LT2 r-m+ *galE* (sensitive to P1 and P22) | [9] | A-151 |

**Strain Relevant genotype Source or references Lab stock**
