# Supplementary material for: mRNA dynamics and alternative conformations adopted under low and high arginine concentrations control polyamine biosynthesis in Salmonella
Source: PLoS Genet. 2019 Feb 11;15(2):e1007646. doi: 10.1371/journal.pgen.1007646 (PMC6386406; doi:10.1371/journal.pgen.1007646)
Supplement: S3 Table — aPrimers used for fragment amplification. bPrimers used to generate mutations. (DOCX) [file pgen.1007646.s012.docx]

**S3 Table. Plasmids**

| Plasmid | Construction | Source |
| --- | --- | --- |
| pGEM4 |  | Lab collection |
| P*speF*-*orf34*AUG-*speF*' | pGEM4 (1636-1637)a | This study |
| P*speF*-*orf34*AAA-*speF*' | pGEM4 (1636-1637)a | This study |
| pRS551 | *lacZ* transcription fusion | [10] |
| pRS552 | *lacZ* translation fusion | [10] |
| P*speF*-*orf34*AUG-*speF'*-*lacZ* | pRS551 (1636-1637)a | This study |
| P*speF*-*orf34*AUG-*speF'*-*'lacZ* | pRS552 (1636-1637)a | This study |
| P*speF*-*orf34*AUG-*lacZ* | pRS551 (1636-1639)a | This study |
| P*speF*-*orf34*AUG-*'lacZ* | pRS552 (1636-1639)a | This study |
| P*speF*-*orf34*AAA-*speF'*-*lacZ* | pRS551 (1636-1637)a (1677-1678)b | This study |
| P*speF*-*orf34*AAA-*speF'*-*'lacZ* | pRS552 (1636-1637)a (1677-1678)b | This study |
| P*speF* -*lacZ* | pRS551 (1636-1898)a | This study |
| P*speF*-orf34rRR-*speF'*-*lacZ* | pRS551 (1636-1637)a (1947-1929)b | This study |
| P*speF*-*orf34*rRR-*speF'*-*'lacZ* | pRS552 (1636-1637)a (1947-1929)b | This study |
| P*speF*-*orf34*fRR-*speF'*-*lacZ* | pRS551 (1636-1637)a (2124-1929)b | This study |
| P*speF*-*orf34* fRR-*speF'*-*'lacZ* | pRS552 (1636-1637)a (2124-1929)b | This study |
| P*speF*-*orf34*KK-*speF'*-*lacZ* | pRS551 (1636-1637)a (1928-1929)b | This study |
| P*speF*-*orf34*KK-*speF'*-*'lacZ* | pRS552 (1636-1637)a (1928-1929)b | This study |
| P*speF*-*orf34*UAA11-*speF'-lacZ* | pRS551 (1636-1637)a (1991-1992)b | This study |
| P*speF*-*orf34*UAA11-*speF'-'lacZ* | pRS552 (1636-1637)a (1991-1992)b | This study |
| P*speF*-*orf34*UAA26-*speF*'-*lacZ* | pRS551 (1636-1637)a (1978-1983)b | This study |
| P*speF*-*orf34*UAA26-*speF*'-*'lacZ* | pRS551 (1636-1637)a (1978-1983)b | This study |
| P*speF*-*orf34*Q34-*speF*'-*lacZ* | pRS551 (1636-1637)a (2047-2048)b | This study |
| P*speF*-*orf34*Q34-*speF*'-*'lacZ* | pRS552 (1636-1637)a (2047-2048)b | This study |
| P*speF*-*orf34*W35-*speF*'-*lacZ* | pRS551 (1636-1637)a (2024-1985)b | This study |
| P*speF*-*orf34* W35-*speF*'-*'lacZ* | pRS552 (1636-1637)a (2024-1985)b | This study |
| pRI | P*tac* | [11] |
| P*tac*-*orf34*-*speF* | pRI (1788-1790)a | This study |
| P*tac*-*orf34*AAA-*speF* | pRI (1788-1790)a (1677-1678)b | This study |
| P*tac*-*orf34*UAA11-*speF* | pRI (1788-1790)a (1991-1992)b | This study |
| P*tac*-*orf34*UAA26-*speF* | pRI (1788-1790)a (1978-1983)b | This study |
| P*tac*-*orf34*rRR-*speF* | pRI (1788-1790)a (1947-1929)b | This study |
| P*tac*-*orf34*KK-*speF* | pRI (1788-1790)a (1928-1929)b | This study |
| P*tac*-*orf34*Q34-*speF*  P*tac*-*orf34*W35-*speF* | pRI (1788-1790)a (2047-2048)b  pRI (1788-1790)a (2024-1985)b | This study  This study |
| P*tac*-*orf34*-Δ(175-182) -*speF* | pRI (1788-1790)a (2449-2450)b | This study |
| P*tac*-*orf34*-Δ(344-352) -*speF* | pRI (1788-1790)a (2451-2452)b | This study |
| P*tac*-*orf34*-Δ(388-400) -*speF* | pRI (1788-1790)a (2453-2454)b | This study |
| P*tac*-*orf34*-Δ(442-449) -*speF*  P*tac*-*orf34*-Δ(170-313) -*speF*  P*tac*-*orf34*-Δ(319-454) -*speF*  pZA31  PL*tetO-1-orf34* | pRI (1788-1790)a (2455-2456)b  pRI (1788-1790)a (2433-2434)b  pRI (1788-1790)a (2435-2436)b  PL*tetO-1*  pZA31 (2368-2369)a | This study  This study  This study  [12]  This study |
