# Supplementary material for: mRNA dynamics and alternative conformations adopted under low and high arginine concentrations control polyamine biosynthesis in Salmonella
Source: PLoS Genet. 2019 Feb 11;15(2):e1007646. doi: 10.1371/journal.pgen.1007646 (PMC6386406; doi:10.1371/journal.pgen.1007646)
Supplement: S4 Table — (A) aPlus (+) and minus (-) strands are indicated. (B) aPlus (+) and minus (-) strands are indicated. (C) aPlus (+) and minus (-) strands are indicated. bAll mutant constructs are based on 616 nt fragment (1636–1637) consisting of PspeF-orf34-speF'-lacZ. cPositions of the mutations or deletions are relative to the transcription start site of speF operon transcript. (DOCX) [file pgen.1007646.s013.docx]

**S4 Table. Oligonucleotides**

**A. Oligonucleotides used for cloning**

| Primer | Primer sequence (5'-3') | Usea |
| --- | --- | --- |
| 1636 | AAGAATTCTATTTTCGGCGTCGCGA | P*-orf34-speF* (EcoRI +) |
| 1637 | AAG GATCCGCCACGTCGATGAAACG | P*-orf34-speF* (BamHI -) |
| 1639 | AAGGATCCCGGGCATTAAAGAAATGAAAGTC | P*-orf34* (BamHI -) |
| 1788 | AAGAATTCGCTTTCAATAAATCAATGAAAGGACCTC | *orf34-speF* (EcoRI +) |
| 1790 | GGGAAGCTTGCCACGTCGATGAAACG | *orf34-speF* (HindIII -) |
| 2212 | CCTGACGTCGAAAGGACCTCAAAATGG | 3' RACE (AatII +) |
| 2213  2368  2369 | CCCAAGCTTGGCCGCTAAGAACAGTGAA  CCCGGTACCCAATAAATCAATGAAAGGACCTC  GCAGAAAAACTACCGGGC | 3' RACE (HindIII –)  *orf34* (KpnI (+)  *orf34* (phosphorylated (-) |

**B. Oligonucleotides used for construction of strains**

| Primer | Primer sequence (5'-3') | Usea |
| --- | --- | --- |
| 1831 | ATTCATTATTTTTAAATAACTTCTAATTTTTATGTCATTATGGGAATTAGCCATGGTCC | Δ*speF* (CR) (cat +) |
| 1832 | TTTCCGTGTTCAATGTCGTCAATGGATAACACGATGGCATGTAGGCTGGAGCTGCTTC | Δ*speF* (CR) (cat -) |
| 1514 | CAGGCCGTTTAAGCGATGATTCACGAGAATTGCTGGCCCGGTCTCAAAATCTCTGATGTTACATTGC | ∆*hisG*46::λRS551/552 |
| 1515 | TCGGCAGTACCAGAATCGAGCTGGCGCCAAGCGCTTTCAGGCTGATATGGTTGATGTCATGTAGCC | ∆*hisG*46::λRS551/552 |

**C. Oligonucleotides used for site directed mutagenesis**

| Primer | Primer sequence (5'-3') | Usea,b,c |
| --- | --- | --- |
| 1677 | AAGAAAATAATAATCGTTTTATGCCCC | AAA (+) |
| 1678 | TTTTGAGGTCCTTTCATTGATTTATTG | AAA (-) |
| 1978 | AAGACTTTCATTTCTTTAATGC | UAA26 (+) |
| 1983 | AACTATTGCGGTGGGC | UAA26 (-) |
| 1991 | GCCCCATTGAAGGCGGACAACG | UAA11(+) |
| 1992 | ATAAAACGATTATTATTTTCCATTTTG | UAA11(-) |
| 1947 | CGACGAACAACGCATATTATGATGTTTG | RR (+) |
| 1928 | AAGAAGACAACGCATATTATGATG | KK (+) |
| 1929 | TATATGGGGCATAAAACGATTATTATT | rRR, fRR, KK (-) |
| 2124 | CGCCGCACAACGCATATTATGATGTTTG | fRR (+) |
| 2047 | AGTAGTTTTTCTGCTACGGGTACTTC | Q34 (+) |
| 2048 | GGGCATTAAAGAAATGAAAGTCGAAACTATTG | Q34 (-) |
| 1888 | GTTTTCTTTAATGCCCGGTAG | CA116,117GT (+) |
| 1889 | AAAGTCGAAACTATTGCGGTG | CA116,117GT (-) |
| 1890 | ACAAAAATGTCAGAATTAAAAATTGC | TG510,511AC (+) |
| 1891 | TCTCTTTATTTCAGGTAATAGCAG | TG510,511AC (-) |
| 2449 | TGTCATTCAGGTCTATTG | Δ(175-182) (+) |
| 2450 | GGAAAGACCTGTATGCTG | Δ(175-182) (-) |
| 2451 | CCTGAATTTCCTGCAACC | Δ(344-352) (+) |
| 2452 | GTTGAGTACTACGATATACAGG | Δ(344-352) (-) |
| 2453 | AAAGCTAATTTACTGATTTTTTTATC | Δ(388-400) (+) |
| 2454 | TAAATCCCAACCCGTCC | Δ(388-400) (-) |
| 2455 | CGGAAGAATCAGTTTCTCATGC | Δ(442-449) (+) |
| 2456  2433  2434  2435  2436  2024  1985  2909  2910 | CAAAGCTATCCCGCTG  TTTCTGCCTGTATATCG  ACCTGTATGCTGAAGTAC  GAATCAGTTTCTCATGCG  AGAAACCGCGATAACTAAAATTC  GGTTTTTCTGCTACGGGTAC  ACCGGGCATTAAAGAAATG  CAGGTCTATTGCCTAAATAACTC  TACCCGTAGCAGAAAAACTAC | Δ(442-449) (-)  Δ(170-313) (+)  Δ(170-313) (-)  Δ(319-454) (+)  Δ(319-454) (-)  W35 (+)  W35 (-)  Δ(154-189) (-)  Δ(154-189) (+) |

**D. Oligonucleotides used for northern blots**

| Primer | Primer sequence (5'-3') | Use |
| --- | --- | --- |
| 1614 | GTTGTCCGCCTTATATGGG | *orf34* |
| 2411 | CATTTTGAGGTCCTTTC | *orf34* carrying RR mutations |
| 459 | GAGACCCCACACTACCATC | 5S rRNA |

**E. Oligonucleotides used for primer extensions**

| Primer | Primer sequence (5'-3') | Use |
| --- | --- | --- |
| 2429 | CATCTCTTTATTTCAGG | To map the region from 344 nt and on |
| 1789 | GGGAAGCTTGGGCGTAAAGGAATTACCGTAC | To map the region from 175 nt and on |
| 1579 | rUrUrCrArCrUrGrUrUrCrUrUrArGrCrGrGrCrCrGrCrArUrGrCrUrC | RNA oligo adapter for 3' RACE |
| 2211 | GGCCGCTAAGAACAGTGAA | Reverse transcription for 3' RACE |
