# Supplementary material for: mRNA dynamics and alternative conformations adopted under low and high arginine concentrations control polyamine biosynthesis in Salmonella
Source: PLoS Genet. 2019 Feb 11;15(2):e1007646. doi: 10.1371/journal.pgen.1007646 (PMC6386406; doi:10.1371/journal.pgen.1007646)
Supplement: S1 Text — (DOCX) [file pgen.1007646.s014.docx]

**S1 Text. Additional references**

1. Larkin MA, Blackshields G, Brown NP, Chenna R, McGettigan PA, McWilliam H, et al. Clustal W and Clustal X version 2.0. Bioinformatics. 2007;23(21):2947-8. Epub 2007/09/10. doi: 10.1093/bioinformatics/btm404. PubMed PMID: 17846036.

2. Dereeper A, Guignon V, Blanc G, Audic S, Buffet S, Chevenet F, et al. Phylogeny.fr: robust phylogenetic analysis for the non-specialist. Nucleic Acids Res. 2008;36(Web Server issue):W465-9. Epub 2008/04/19. doi: 10.1093/nar/gkn180. PubMed PMID: 18424797; PubMed Central PMCID: PMCPMC2447785.

3. Guindon S, Dufayard JF, Lefort V, Anisimova M, Hordijk W, Gascuel O. New algorithms and methods to estimate maximum-likelihood phylogenies: assessing the performance of PhyML 3.0. Syst Biol. 2010;59(3):307-21. Epub 2010/03/29. doi: 10.1093/sysbio/syq010. PubMed PMID: 20525638.

4. Whelan S, Goldman N. A general empirical model of protein evolution derived from multiple protein families using a maximum-likelihood approach. Mol Biol Evol. 2001;18(5):691-9. doi: 10.1093/oxfordjournals.molbev.a003851. PubMed PMID: 11319253.

5. Bateman A, Coin L, Durbin R, Finn RD, Hollich V, Griffiths-Jones S, et al. The Pfam protein families database. Nucleic Acids Res. 2004;32(Database issue):D138-41. doi: 10.1093/nar/gkh121. PubMed PMID: 14681378; PubMed Central PMCID: PMCPMC308855.

6. Gorodkin J, Heyer LJ, Brunak S, Stormo GD. Displaying the information contents of structural RNA alignments: the structure logos. Comput Appl Biosci. 1997;13(6):583-6. PubMed PMID: 9475985.

7. Schneider TD, Stephens RM. Sequence logos: a new way to display consensus sequences. Nucleic Acids Res. 1990;18(20):6097-100. PubMed PMID: 2172928; PubMed Central PMCID: PMCPMC332411.

8. Hammarlöf DL, Liljas L, Hughes D. Temperature-sensitive mutants of RNase E in Salmonella enterica. J Bacteriol. 2011;193(23):6639-50. Epub 2011/09/23. doi: 10.1128/JB.05868-11. PubMed PMID: 21949072; PubMed Central PMCID: PMCPMC3232887.

9. Bullas LR, Ryu JI. Salmonella typhimurium LT2 strains which are r- m+ for all three chromosomally located systems of DNA restriction and modification. J Bacteriol. 1983;156(1):471-4. PubMed PMID: 6352690; PubMed Central PMCID: PMCPMC215113.

10. Simons RW, Houman F, Kleckner N. Improved single and multicopy lac-based cloning vectors for protein and operon fusions. Gene. 1987;53(1):85-96. PubMed PMID: 3596251.

11. Opdyke JA, Kang JG, Storz G. GadY, a small-RNA regulator of acid response genes in Escherichia coli. J Bacteriol. 2004;186(20):6698-705. doi: 10.1128/JB.186.20.6698-6705.2004. PubMed PMID: 15466020; PubMed Central PMCID: PMCPMC522195.

12. Lutz R, Bujard H. Independent and tight regulation of transcriptional units in Escherichia coli via the LacR/O, the TetR/O and AraC/I1-I2 regulatory elements. Nucleic Acids Res. 1997;25(6):1203-10. PubMed PMID: 9092630; PubMed Central PMCID: PMCPMC146584.
